# Indomuscone-Based Sterically Encumbered Phosphines as Ligands for Palladium-Catalyzed Reactions

**DOI:** 10.1021/acs.joc.3c00314

**Published:** 2023-04-07

**Authors:** Francisco Garnes-Portolés, Sergio Sanz-Navarro, Jordi Ballesteros-Soberanas, Ana Collado-Pérez, Jorge Sánchez-Quesada, Estela Espinós-Ferri, Antonio Leyva-Pérez

**Affiliations:** † Instituto de Tecnología Química, Universitat Politècnica de València-Consejo Superior de Investigaciones Científicas, Avda. de los Naranjos s/n, 46022 Valencia, Spain; ‡ International Flavours & Fragrances Inc., Avda Felipe Klein 2, 12580 Benicarló, Castellón, Spain

## Abstract

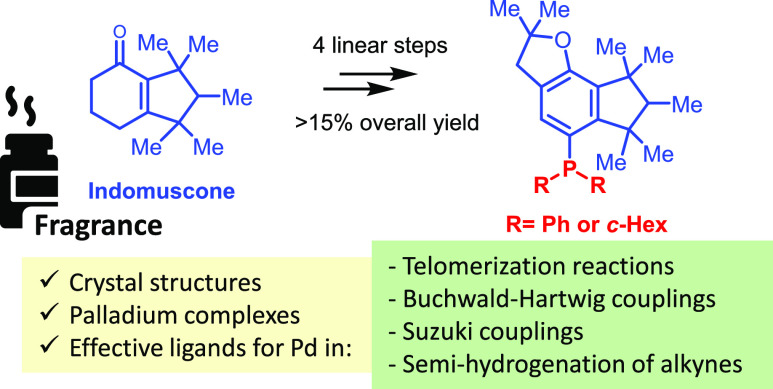

The fragrance compound indomuscone is used here as a scaffold to prepare two different sterically hindered phosphines, one aromatic and another alkylic, in good yields, after four synthetic steps. The new phosphines show enhanced electronic and steric properties when compared to benchmark commercial phosphine ligands, which is reflected in the catalytic results obtained for representative palladium-catalyzed reactions such as the telomerization reaction, the Buchwald–Hartwig and Suzuki cross-coupling reactions of chloroaromatic rings, and the semi-hydrogenation reaction of an alkyne. In particular, the indomuscone-based aromatic phosphine ligand leads to the highest selectivity for the tail-to-head telomerization product between isoprene and methanol, while the indomuscone-based alkylic phosphine ligand shows extraordinary similarities with the Buchwald-type SPhos phosphine ligand.

## Introduction

Phosphines are very active ligands for palladium-catalyzed organic reactions, which include cross-coupling reactions,^1^ hydrogenations,^2^ and hydro(alkoxy)formylations,^2,3^ to name a few.^4^ In particular, sterically encumbered phosphines are considered privileged ligands in palladium-catalyzed reactions such as the telomerization reaction,^5^ the Buchwald–Hartwig^6^ and Suzuki^7^ cross-coupling reactions, the semi-hydrogenation reaction of alkynes,^8^ and others.^9^ The rationale behind the high catalytic activity of these phosphines with palladium lies, on one hand, on the protection of the metal site during the catalytic cycle, which avoids typical palladium deactivation processes such as reduction or aggregation, and, on the other hand, on the formation of *ipso* carbon-palladium bonds that pre-activates the catalytic site for reagent coordination.^10^ In addition, the constrained local environment provided by the phosphine around the metal site directs the selectivity of the reaction toward the desired product in many cases, and the steric shield around the P atom increases the stability toward air, which are both additional advantages in catalysis.^1^

**Figure 1:**
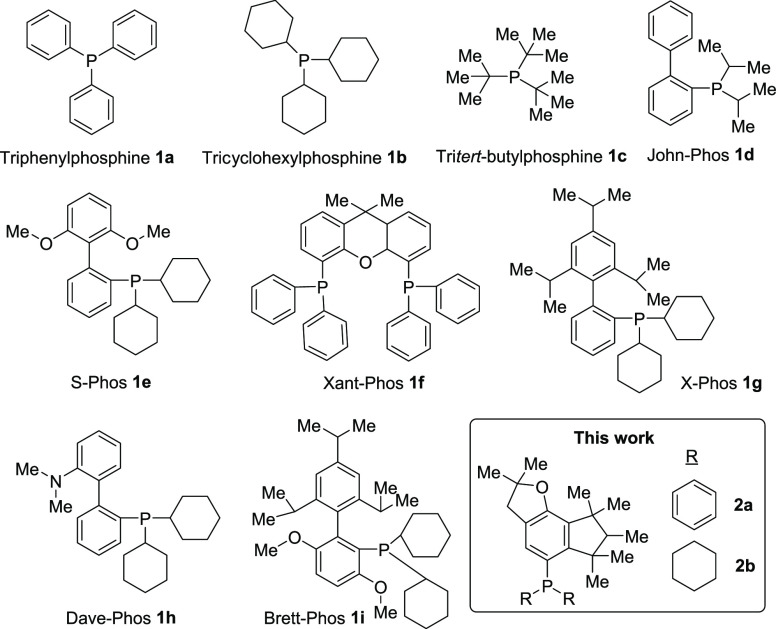
Commercially available phosphines used in metal catalysis as ligands (**1a**–**1i**), sterically encumbered or not, and the phosphine ligands reported in this work (**2a** and **2b**).

Figure 1 shows typical phosphine ligands (**1a**–**1d**) and representative examples of sterically hindered phosphines (**1e**–**1i**), most of them currently under industrial use as ligands for palladium-catalyzed reactions. It can be seen that a common, prominent feature of these ligands is the combined use of cyclohexyl and aromatic rings with methyl multisubstituted tertiary and quaternary carbon atoms (i.e., isopropyl groups), which, together, generate the desired sterically crowded structure around the palladium site. It is worthy commenting here that small structural changes in the phosphine produce dramatic changes in the final catalytic activity of the corresponding palladium complex.^1,6^ For all these reasons, the search for new sterically hindered phosphines is of much interest, and even machine learning is being used for that.^11^ However, as new phosphines are discovered, more sophisticated starting materials and synthetic routes are required, which translates into long and expensive synthetic protocols. In this way, the final price of the ligand can be even superior to the palladium atom itself. Thus, the search for precursors of phosphine ligands that contain new sterically encumbered structural features and, at the same time, are cheap and widely available is of interest from both scientific and economic point of views. Here, we report two new phosphine ligands based on a widely available and cheap fragrance compound, as shown in Figure 1 (**2a** and **2b**).

## Results and Discussion

### Synthesis of Phosphines **2a** and **2b**

**Figure 2:**
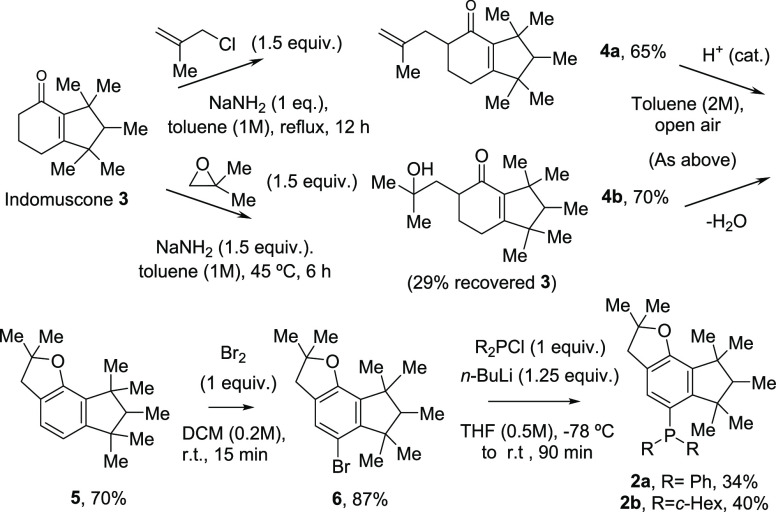
Synthesis of phosphines **2a** and **2b**. Yields refer to isolated products.

The synthesis of the sterically encumbered phosphines **2a** and **2b** is shown in Figure 2. The precursor is the fragrance compound indomuscone **3** (commercial name Cashmeran), which is produced in multiton amounts per year with a price <0.05 $/g. This widely available compound is easily transformed in two simple steps to the polycyclic compound **5** in good yields, either through the methallyl **4a** or the hydroxyl **4b** derivative. Both intermediates are obtained after deprotonation in the α-position of **3** with solid sodium amide (NaNH_2_) and alkylation of the in situ-generated enolate with the corresponding electrophiles. Any other deprotonation agent tested, including sodium methoxide (NaOMe, solid or in THF solution), sodium or potassium *tert-*butoxide (Na or KO^t^Bu, solids or in solution), NaH, or lithium reagents were completely ineffective for the reaction. We tentatively ascribe the uniqueness of NaNH_2_ as a base for this reaction to the release of NH_3_ as a gas, which avoids the reversal of the equilibrium. Despite **4b** being produced with complete selectivity and 29% of starting material could be recovered after reaction by column chromatography separation, the lower price of the methallyl electrophile made us to choose this reaction for scaling up.

The aromatization step to generate **5** can be carried with different acid catalysts, including typical soluble organic acids such as *p*-toluenesulfonic acid (pTsOH) and methylsulfonic acid (MSA), and also solid acids such as zeolites and sulfonic resins (see Tables S1 and S2 in the Supporting Information), achieving a similar yield of 70% for pTsOH and H-Beta zeolite under the optimized conditions (Tables S2–S6 and Figure S1). Toluene is the best solvent for the transformation (Tables S5 and S6). The reaction is run under “open flask” conditions to use air as an oxidant for the dehydrogenation step, which gives access to the desired aromatic ring. Otherwise, the reaction stops in the corresponding dienes, detected by GC–MS, which suffer from isomerizations and other undesired transformations.

**Figure 3:**
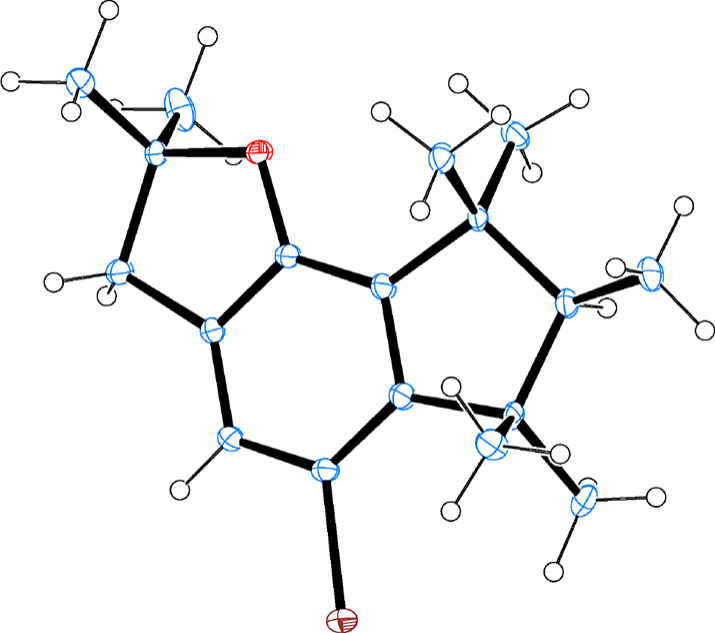
Crystal structure of the intermediate bromoderivative **6** obtained by single-crystal XRD. Ellipsoids represent a 30% probability. Color code: Br in brown, O in red, C in blue, and H in white.

Compound **5** features a sterically shielded indane ring that can be easily brominated in the *ortho* position to the penta-substituted methyl cyclopentane cycle, by the inductive action of the ether substituent in the *para* position, to yield **6** in high yield in just a 15 min reaction time at room temperature. Multigram amounts of compound **6** were easily obtained as yellowish crystals after work-up and removal of volatiles under vacuum, without requiring any crystallization technique, and Figure 3 shows the resolved crystalline structure by monocrystal X-ray diffraction (XRD, see the Supporting Information for a summary of crystallographic data). The sterically crowded environment imparted by the aromatic indomuscone core can be seen together with the Br atom substitution, which, in principle, leaves room for the introduction of a phosphine moiety.

**Figure 4:**
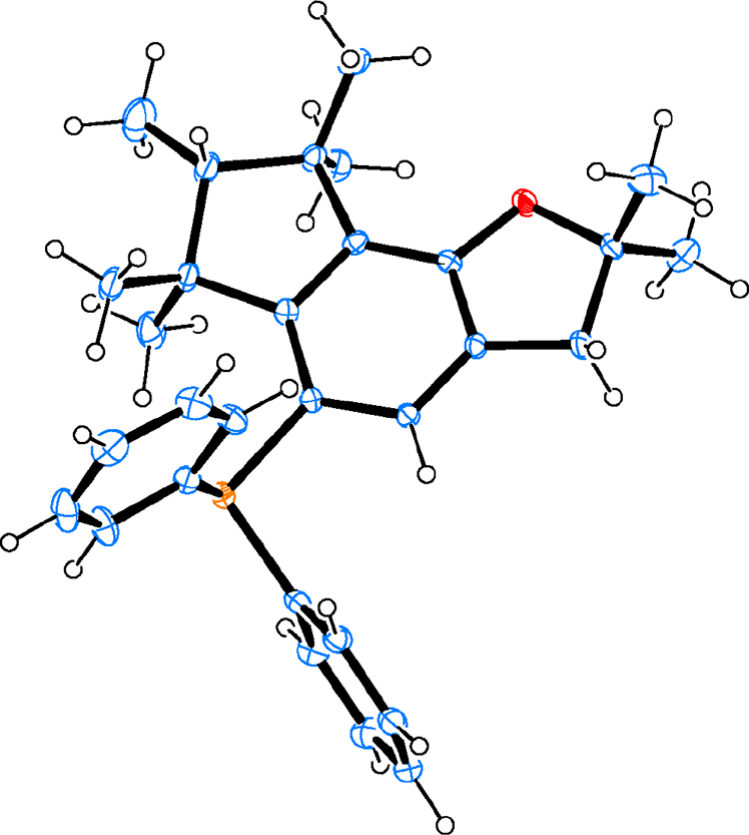
Crystal structure of phosphine **2a** obtained by single-crystal XRD. Ellipsoids represent a 30% probability. Color code: P in orange, O in red, C in blue, and H in white.

Thus, with multigram quantities of **6** in hand, we proceeded to couple **6** with two selected chlorophosphines of general structure ClPR_2_, in this case, the representative phenyl (R = Ph) and cyclohexyl (R = *c*-hex) substituent groups, under typical reaction conditions.^12^ For the former, the final targeted phosphine **2a** was isolated by column chromatography in moderate yields and, as precursor **6**, spontaneously crystallized at room temperature, to give white crystals. The overall yield after four linear steps is 16%. Figure 4 shows the crystalline structure of phosphine **2a**, where the sterically encumbered coordination shell around the P atom can be observed.

**Figure 5:**
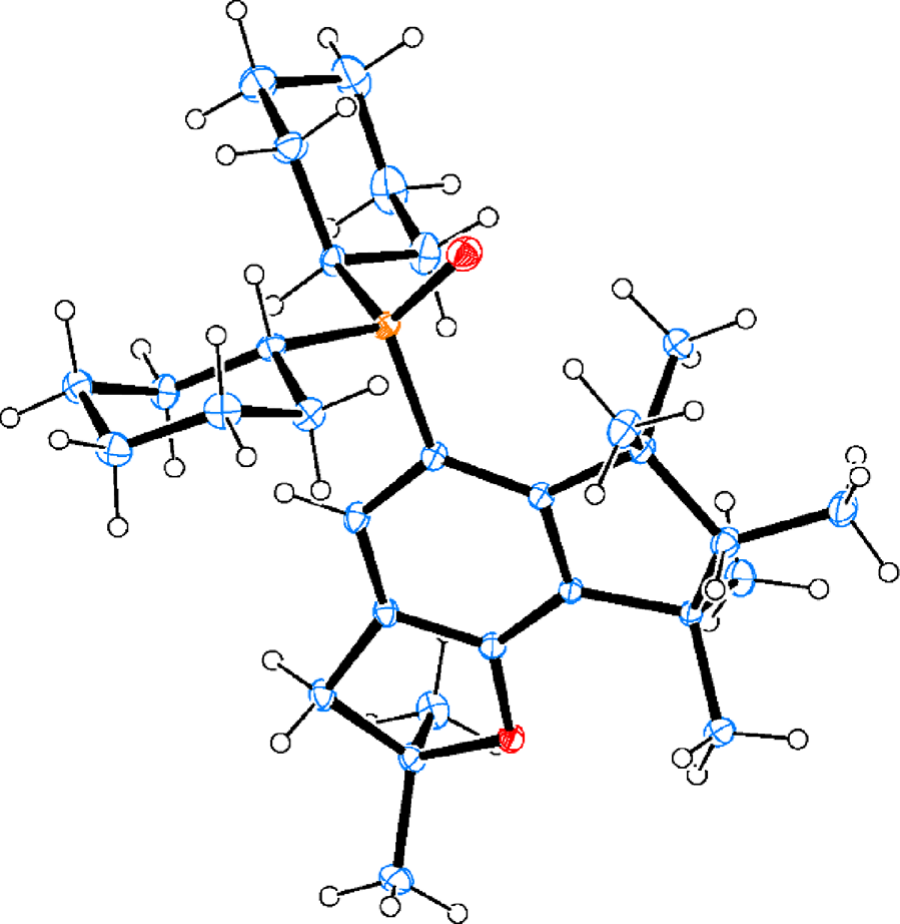
Crystal structure of phosphine oxide **2b-oxide** obtained by single-crystal XRD. Ellipsoids represent a 30% probability. Color code: P in orange, O in red, C in blue, and H in white.

In contrast to **2a**, it was not possible to obtain crystals of **2b** after isolation by column chromatography and recrystallization tests; however, the crystal structure of the oxidized form (**2b-oxide**) could be obtained. Figure 5 shows the crystal structure of **2b-oxide**, which is similar to **2a**, with the P atom surrounded by the aromatic indomuscone and the two cyclohexyl substituents groups.

With the crystal structure in hand, the electronic and steric properties of the new phosphines were calculated and compared with the commercial phosphines **1a**–**e** (Table S7). The electronic and steric properties were assessed by the vibrational frequency of the carbonyl stretch of the corresponding Ni(CO)_3_L complex (2056.1–2073.0 cm^–1^),^13^ which directly correlates to the phosphine lone pair charge density^14^ and the Tolman cone angle, respectively.^13^ It is worth noting to comment here that Tolman cone angle values often overestimate steric hindrance in elaborated asymmetric phosphines, bidentate ligands, and *N*-heterocyclic carbenes, and that other values such as percent buried volume (*V*_bur_)^15a^ and ligand repulsive energy parameter (ER)^15b^ can be more suitable here. However, for the sake of simplification, we will use the well-established Tolman parameter in a first approximation. It can be seen that phosphine **2a** shows an electronic value similar to triphenylphosphine **1a** (2067 vs 2069 cm^–1^) but a completely opposed steric value (195 vs 145°), 40° higher and in the range of very hindered phosphines. The phosphine **2b** values are 2058 cm^–1^ and 205° (inferred from **2b-oxide**), which denotes a much higher electron donating capability and even higher steric hindrance than **2a**.

Notice that the values for **2b** are extraordinarily similar to SPhos **1e**. The combined electronic and steric properties of phosphines **2a** and **2b** make them attractive for use as ligands in catalysis. In addition, the unique polysubstituted tricyclic structure of aromatic indomuscone may confer to the new phosphine ligands an enhanced catalytic action. Notice here that the final R groups in **2** can be chosen for a plethora of other available chlorophosphines (R_2_PCl); thus, this family of phosphines could be easily widened if desired.

### Synthesis of the Palladium–Phosphine **2a** Complex

**Figure 6:**
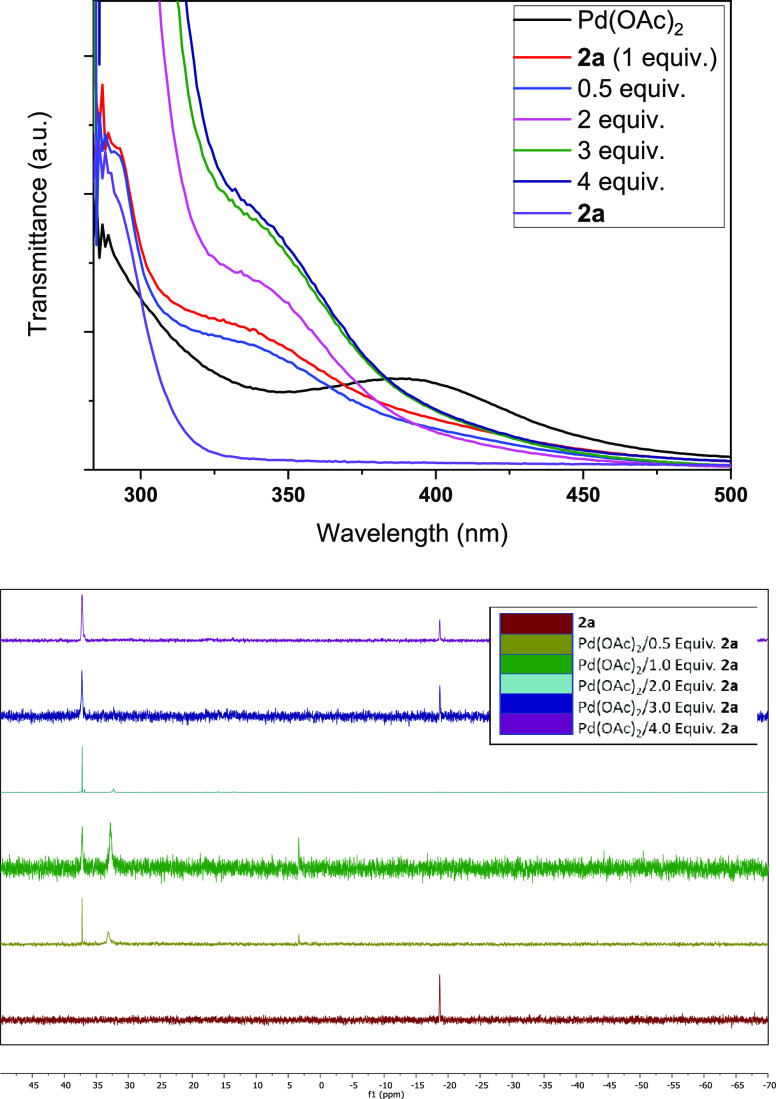
Titration of palladium acetate with phosphine **2a** by UV–vis (top) and ^31^P-NMR (bottom), in methanol solution.

The titration of palladium acetate with phosphine **2a** was accomplished in the methanol solvent by UV–vis and ^31^P-NMR. Figure 6 (top) shows the UV–vis results after adding from 0.5 to 4 equiv of **2a**, and it can be seen that the typical broad absorption band of palladium acetate at ∼380 nm (black curve) disappears after adding two or more equivalents of **2a** to give a new single absorption band at ∼340 nm (i.e. pink curve). This band is more pronounced for 2 equiv of **2a**, although it is not discarded compared to 3 equiv of **2a** that are filling the coordination sphere of the palladium cation, according to these spectra.

The corresponding ^31^P-NMR measurements in Figure 6 (bottom) show that the original peak of phosphine **2a** at ∼20 ppm (red line) transforms into a new single peak at ∼40 ppm when 2 equiv of **2a** is added to palladium acetate (light blue line), while lower amounts of **2a** give a mixture of signals. These results indicate that a 1:2 Pd:phosphine complex is being formed. Indeed, the signal at∼40 ppm remains and coexists with the original peak of phosphine **2a** at ∼20 ppm when >2 equiv of **2a** is added, indicating that [Pd(**2a**)_2_]^2+^ is a stable species that does not admit a third phosphine ligand in the palladium coordination shell, in accordance with the sterically encumbered structure of **2a**. Thus, we must conclude here that Pd(**2a**)_2_(OAc)_2_ is the more plausible complex formed after mixing both species.

With these results in hand, we stirred 1 equiv of palladium acetate and 2 equiv of phosphine **2a** in MeOH at 50 °C for 5 min, and then, we concentrated and obtained an orange solid, in which analytical results perfectly matched those expected for Pd(**2a**)_2_(OAc)_2_, including the observed UV–vis and ^31^P NMR signals, and also ICP-OES and elemental analysis values. Particularly informative are the UPLC-HRMS results (Figure S2), where the main peak of the spectrum corresponds to the [Pd(**2a**)_2_(OAc)]^+^ cation, after losing one of the acetates, with the associated isotopic distribution for one Pd atom and the exact mass of this cationic complex. Minor signals corresponding to free phosphine **2a**, oxidized palladium complex, and complex dimers can also be observed. We could obtain some small crystals after extensive crystallization tests; however, these crystals proved unsuitable for XRD.

The corresponding study with phosphine **2b** was not carried out by the tendency to oxidize of the alkyl phosphine; however, in a first approximation, we can expect a stable 1:2 Pd:phosphine complex.

### Catalytic Results

#### Telomerization Reaction

The telomerization reaction is an industrial reaction catalyzed by palladium phosphine complexes in solution, typically not only with triphenylphosphine ligands **1a** but also with other phosphines.^5^ A 1:2 Pd: phosphine catalytic ratio is very common in this reaction, and sterically encumbered phosphines have been studied.^16^ Thus, we tested the telomerization reaction with the new phosphine ligands **2a** and **2b**, and the results are shown in Table 1.

**Table 1:**
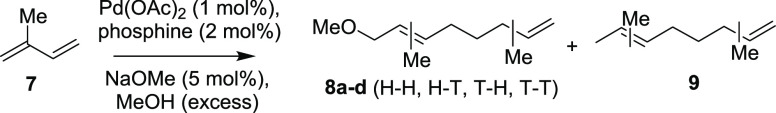
Catalytic Results for the Telomerization Reaction of Isoprene **7** and MeOH Catalyzed by 1:2 Pd(OAc)_2_-Phosphine Complex Catalysts

entry	phosphine	MeOH (equiv)	conv. (%)^a^	yield to **8** (%)	ratio (**8a**–**d**)
1	**1a**	12	100	95	10/4/67/19
2	**2a**	12	100	98	3/-/95/2
3	**1a**	5	95	91	18/3/60/19
4	**2a**	5	98	92	5/-/93/2
5	**1a**	2	95	68	25/5/41/29
6	**2a**	2	96	72	7/-/88/5
7	**1c**	12	76	2	25/-/60/15
8	**1e**	12	85	5	30/-/40/30
9	**2b**	12	100	70	10/-/70/20

a GC results. Mass balance completed with byproducts **9**.

Isoprene **7** and MeOH were used as substrates to assess not only the catalytic activity of the different phosphine ligands but also the selectivity toward the different products, which in this case arise from the head/tail potential couplings (Figure S3, top).^17^ The catalytic results show that phosphine **2a** outperforms triphenylphosphine **1a** not only in catalytic activity but also, particularly, in selectivity, under the indicated reaction conditions (compare entries 1 and 2). The yield to the tail-to-head telomerization product **8c** with ligand **2a** is 94%, while **1a** gives 65%. The selectivity of the reaction can be clearly seen by ^1^H-NMR spectroscopy (Figure S3, bottom). Remarkably, an 85% yield of **8c** is obtained when decreasing the excess of MeOH from 12 to 5 equiv with ligand **2a** (entries 3 and 4) and a good yield of **8c** is still obtained with just 2 equiv of MeOH, in contrast to ligand **1a** (entries 5 and 6). Alkyl phosphine ligands such as **1c** and **1e** are barely active to produce telomerization products **8a**–**d**, giving only byproducts **9** (entries 7 and 8). However, the alkyl phosphine ligand **2b** gives a 70% yield of products **8a**–**d** with a 70% selectivity to product **8c** (entry 9), clearly improving the results with other alkyl phosphines.

**Figure 7:**
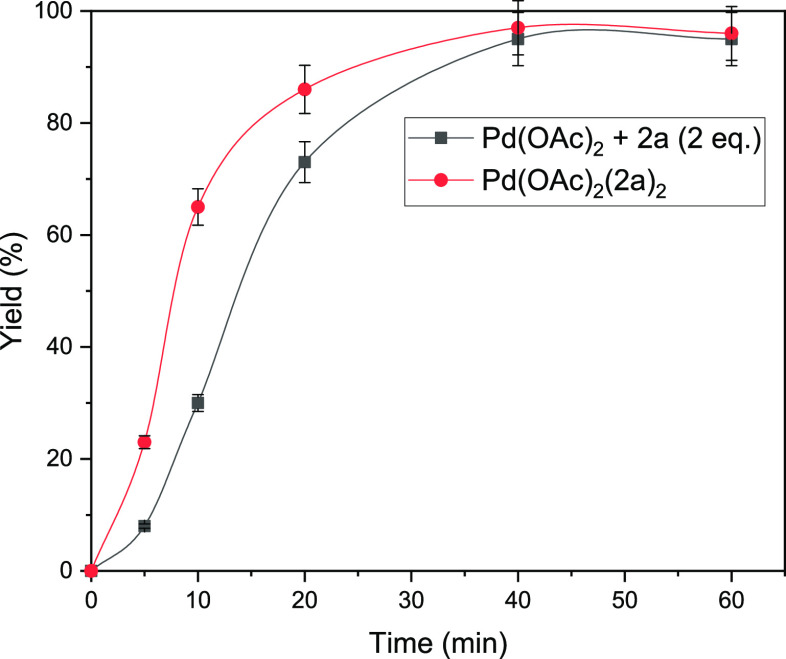
Kinetic plot for the telomerization reaction of isoprene **7** and MeOH catalyzed by 1:2 Pd(OAc)_2_-phosphine complex catalysts, either adding by separating the Pd source and the ligand (gray squares) or pre-forming the complex Pd(**2a**)_2_(OAc)_2_ (red circles). GC results. Error bars account for a 5% uncertainty.

Figure 7 shows the kinetic results for the telomerization reaction under optimized conditions, either adding Pd(OAc)_2_ and ligand **2a** by separating from the beginning in a 1:2 molar ratio or adding the isolated complex Pd(**2a**)_2_(OAc)_2_. The results show an induction time when the complex is prepared in situ and the disappearance of this induction time when the isolated complex is used, supporting that Pd(**2a**)_2_(OAc)_2_ is the active pre-catalyst of the reaction.^18^ It has been proposed that the Pd species in the catalytic cycle are in reduced form, and that they just have one or none phosphine ligands;^5a^ thus, the observed acceleration by preforming Pd(**2a**)_2_(OAc)_2_ could be due to a facile reductive elimination by the phosphine and OAc ligands.

Different alcohols, except those sterically impeded such as isopropanol, engage well in the reaction, and the tail-to-head isomer is systematically the major isomer (Figure S4). These results showcase the superiority of the fragrance-based phosphines **2a** and **2b** for the telomerization reaction with respect to state-of-the-art phosphine ligands, at least in selectivity to the tail-to-head isomer under the reaction conditions tested.

#### Buchwald–Hartwig Cross-Coupling Reaction

The Buchwald–Hartwig coupling reaction is also a well-implemented reaction procedure in fine chemical synthesis to generate new carbon–nitrogen bonds, and Buchwald-type phosphines such as **1e** and **1g**–**i** are privileged ligands for this palladium-catalyzed reaction.^1,6,19^Table 2 shows the catalytic results for the coupling between *p*-chloroanisole **10** and morpholine **11** under typical reaction conditions.^6a^ Chloroaromatics are among the most difficult substrates to activate in this reaction; thus, chloroderivative **10** was used as the starting material. Bis-palladium tris-dibenzylacetonate [Pd_2_(dba)_3_] was employed here as a palladium source since palladium acetate proved inactive (see below).

**Table 2:** Catalytic Results for the Coupling between *p*-Chloroanisole **10** and Morpholine **11** under Typical Reaction Conditions, Catalyzed by Different Pd-Phosphine Complex Catalysts

entry	Pd (mol %)	phosphine (mol %)	conv. (%)^a^	select. to **12** (%)
1	2.5	**1e** (5)	100	98
2		**1a** (5)	2	100
3		**1c** (5)	60	90
4		**2a** (5)	2	50
5		**2b** (5)	40	99
6	5	**2b** (10)	80	92
7		**2b** (5)	38	82
8	2.5	**2b** (7.5)	85	98
9		**2b** (10)	86	97
10^b^		**2b** (7.5)	8	97
11^c^		**2b** (7.5)	38	90

a GC results. Mass balance completed with anisole **13**.

b Pd(OAc)_2_ instead of Pd_2_(Dba)_3_.

c Pd(Dba)_2_ instead of Pd_2_(Dba)_3_.

The catalytic results show that, as expected, SPhos ligand **1e** gives a 98% yield for the coupling product **12** (entry 1), while the pure aromatic triphenylphosphine ligand **1a** is merely inactive for the reaction (entry 2), and alkyl phosphine **1c** gives moderate yields of **12** (54%) with good selectivity (90%, entry 3). In accordance, we found that the aromatic phosphine **2a** is completely inactive for the reaction (entry 4) while the alkyl phosphine **2b** is moderately active (40%) and completely selective toward **12** (99%, entry 5). Further optimization for ligand **2b** shows that, by increasing the amount of Pd_2_(dba)_3_ and ligand **2b**, high yields of **12** can be obtained (>80%, entries 6–9). Palladium acetate is barely active for the reaction (entry 10), while palladium dibenzylacetonate gives lower yields (35%, entry 11).

The results above indicate that phosphine **2b** can be used as a ligand for the Buchwald–Hartwig coupling of chloroaromatics and morpholine **11**, under optimized reaction conditions. Indeed, other chloroderivatives engage well in the reaction (Figure S5). These results, together, confirm the potential use of phosphine **2b** as a ligand in Buchwald–Hartwig cross-coupling reactions.

#### Suzuki Cross-Coupling Reaction

The Suzuki coupling of chloroaromatic derivatives with arylboronic acids is also a challenging reaction catalyzed with high efficiency for palladium complexes of sterically encumbered phosphines.^7,20^Table 3 shows the catalytic results for the Suzuki coupling between *p*-chlorotoluene **14** and phenylboronic acid **15**, under typical reaction conditions. As expected, SPhos **1e** gives the best results among the commercial phosphine ligands tested when using 2.5 mol % of the palladium catalyst (entries 1–3), with a 61% yield of product **16** in our hands. However, at these catalyst loadings, phosphines **2a** and **2b** proved to be low efficient (<20% yield, entries 4 and 5). Toluene **17** was found as the main byproduct of the reaction, and the homocoupling of phenylboronic acid **15** was found to be marginal by an independent reaction test with phosphine ligand **2b**. With these data in hand, the selectivity for cross-coupling product **16** with the phosphine ligand **2b** (80%) was the highest among all phosphines tested; thus, we doubled the amount of catalyst to improve the yield. With these new conditions, a 32% yield of **16** could be obtained, which is anyway still far from the results with SPhos **1e**. The results could be expanded to other chloroaromatic derivatives and arylboronic acids (Figure S6).

**Table 3:**
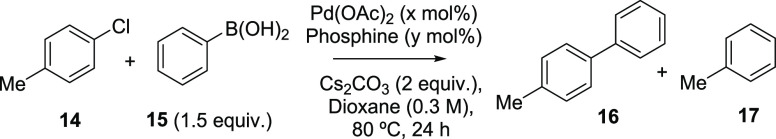
Catalytic Results for the Cross-Coupling Reaction between *p*-Chlorotoluene **14** and Phenylboronic Acid **15** under Typical Reaction Conditions, Catalyzed by Different Pd-Phosphine Complex Catalysts

entry	Pd (mol%)	phosphine (mol%)	conv. (%)^a^	select. to **16** (%)
1	Pd(OAc)_2_ (2.5)	**1e** (5)	83	74
2		**1a** (5)	27	5
3		**1c** (5)	60	70
4		**2a** (5)	20	10
5		**2b** (5)	25	80
6^b^		**2b** (10)	16	49
7	Pd(OAc)_2_ (5)	**2a** (10)	40	12
8		**2b** (10)	50	65
9^c^		**2b** (10)	48	64
10		**2b** (15)	55	63

a GC results. Mass balance completed with toluene **17**.

b Pd_2_(dba)_3_ instead of Pd(OAc)_2_.

c NaO^t^Bu instead of Cs_2_CO_3_.

In any case, these results confirm the better performance of the alkyl phosphine **2b** with respect to the aromatic phosphine **2a** in cross-coupling reactions, in accordance with the results observed with commercial phosphines. It is also worthy to comment here that the concordance in catalytic activity between commercial phosphines and the new phosphines **2a** and **2b** is also observed in the telomerization reaction, in this case with the aromatic phosphines as the more active ligands. We have to add that we tested the above commented couplings with Ni(OAc)_2_ instead of palladium; however, the desired products were not found.

#### Semi-Hydrogenation Reaction of 3-Methyl-1-pentyn-3-ol **18**

**Figure 8:**
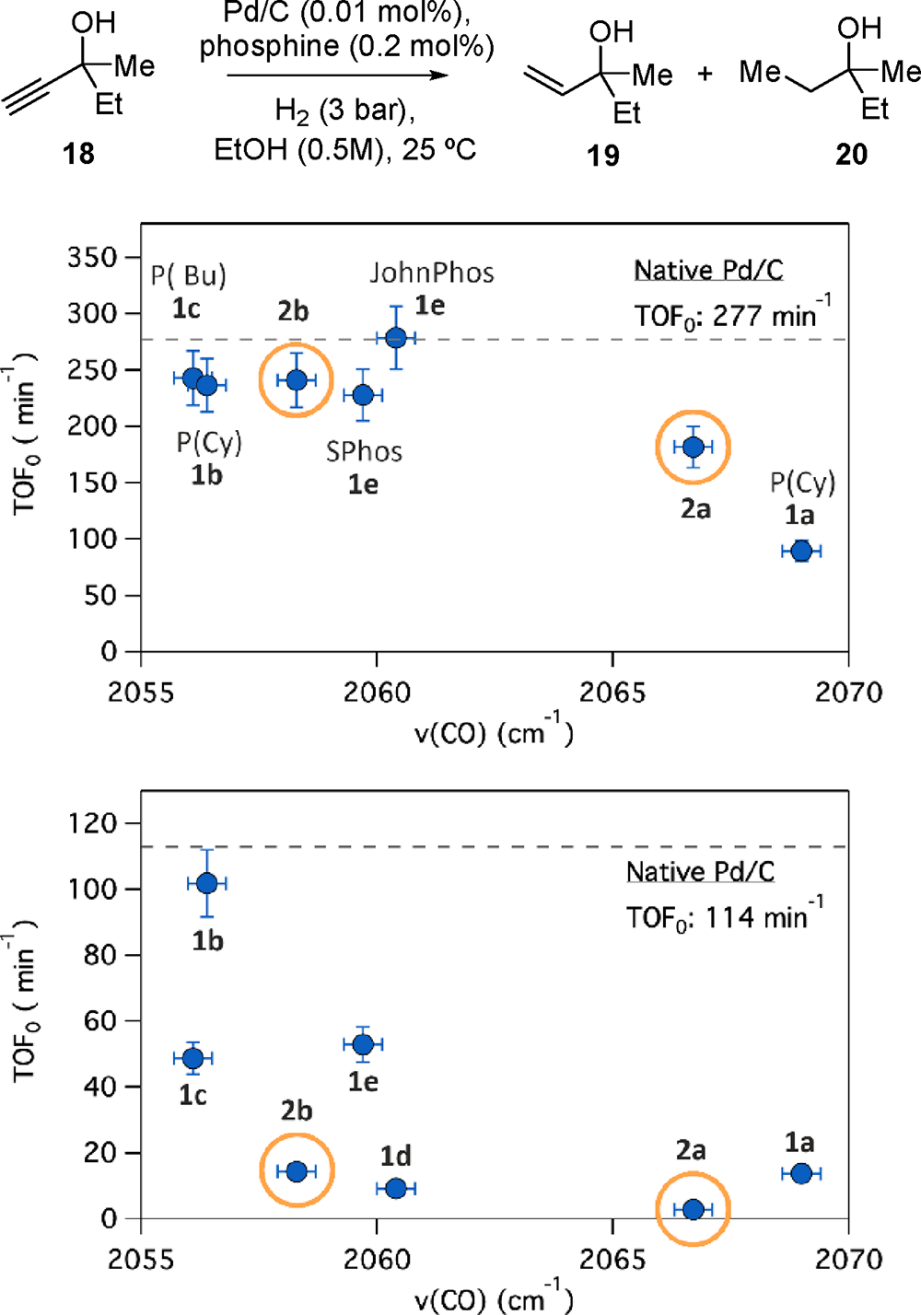
Top: correlation between turnover frequencies in the alkyne to alkene hydrogenation reactions and the electronic properties of the phosphines, expressed in terms of ν(CO) (cm^–1^) of the corresponding Ni(CO)_3_(PR_3_) complex. Bottom: same values for the alkene to alkane transformation. The reactions were performed with Pd/C (1 wt %, 0.01 mol %) at 3 bar and 1:20 molar ratio Pd:phosphine. Vertical error bars represent ±10% uncertainty, and horizontal error bars represent the ±0.3 cm^–1^ uncertainty reported.^13a,14^ The phosphines synthesized in this work (**2a** and **2b**) are encircled. GC results.

The selective semi-hydrogenation of alkynes to *cis* alkenes catalyzed by palladium nanoparticles is an important reaction in the industrial synthesis of nutraceuticals, pheromones, and vitamins, among others.^21^ Supported bare palladium nanoparticles are not selective, and thus, they are treated with modifiers to achieve the desired selectivity. The catalyst of choice here is the classical Lindlar catalyst, composed of Pd nanoparticles supported on CaCO_3_ and poisoned with lead and quinoline.^22^ Alternatively, different supported palladium nanoparticles can be poisoned with other agents such as sulfides,^23^ amines,^24^ and phosphines.^8,25^ Thus, we tested the action of new phosphines **2a** and **2b** in the commercial solid Pd/C, an unselective catalyst for the semi-hydrogenation reaction of alkynes. This solid is composed by palladium nanoparticles of ∼10 nm average size, as assessed by dark field scanning transmission electron microscopy (DF-STEM; Figure S7). Figure 8 shows the catalytic results for the semi-hydrogenation reaction of 3-methyl-1-pentyn-3-ol **18**, a representative industrial reaction.

The semi-hydrogenation reaction of **18** proceeds with the best selectivity if the Pd/C catalyst (0.01 mol %) is treated with JohnPhos **1d**,^26^ SPhos **1e**,^10a^ or phosphine **2b** (0.02 mol %) due to their ability to prevent the alkene to alkane reaction while enabling the desired semi-hydrogenation of **18**. Phosphines **2a** and **1a** are selective as well but hinder the semi-hydrogenation rates. In contrast, phosphine ligands **1b**–**c** are much less selective for the alkene product **19**, giving high amounts of alkane **20** (Figure 8, bottom). This effect is observed at different H_2_ pressures (Figure S8).^27^ These results illustrate again the similarity of phosphine **2b** with Buchwald-type phosphines. In addition, it must be noticed here that the aromatic phosphine **2a** is, in any case, more active than triphenylphosphine **1a**, thus further confirming the apparent superiority of **2a** to **1a** due to the larger volume and higher inductive effect of phosphine **2a**’s Buchwald-type ligand.^27c^

## Conclusions

The synthesis of the indomuscone-based, sterically crowded phosphines **2a** and **2b** has been accomplished in good yields after four synthetic steps. The new phosphine ligands have been characterized by different techniques including X-ray diffraction and show combined electronic and Tolman cone angle values, which differ significantly from most of the commercial phosphines regularly used as ligands in metal catalysis. The Pd(**2a**)_2_(OAc)_2_ complex has also been prepared and characterized and shows enhanced catalytic activity and selectivity to the tail-to-head product **8c** during the telomerization of isoprene **7** in MeOH. Phosphine **2b** shows catalytic similarities to the Buchwald-type SPhos phosphine ligand **1e** during the Buchwald–Hartwig and Suzuki cross-coupling reactions and the semi-hydrogenation reaction of alkyne **18**. These results provide new phosphine ligands for metal catalysis based on a sterically hindered but widely available fragrance compound and the possibility of generating a whole phosphine family since only the phenyl and cyclohexyl derivatives have been employed here.

## Experimental Methods

### Materials

Glassware was dried in an oven at 175 °C before use. Cyclization reactions were performed in vials or round bottom flasks equipped with a magnetic stirrer and open to the air. Reagents and solvents were obtained from commercial sources and were used without further purification unless otherwise indicated. All zeolites are commercially available from Zeolyst. Alkyne **18** and phosphines were purchased from Merck-Millipore Sigma (95–99% purity), except for **1e** (98% purity), purchased from Abcr. Pd/C (1 wt %) was purchased from Merck-Millipore Sigma and used as received. Alkene **19** was independently synthesized by selectively hydrogenating **18** under 1 bar of H_2_ with colloidal Pd nanoparticles supported on TiS (c-Pd/Tis, BASF) at room temperature. Compounds **2a**, **2b-oxide**, and **6** were assigned in the Cambridge Structural Database with deposition numbers CCDC 2240260, 2240261, and 2240259, respectively.

### Physical Techniques

Products were characterized by GC–MS, ^1^H-NMR_,_^31^P-NMR, ^13^C-NMR, and DEPT (distortionless enhancement by polarization transfer). Gas chromatographic (GC) analyses were performed in an instrument equipped with a 25 m capillary column of 5% phenylmethylsilicone, and *n*-dodecane was used as an external standard. Gas chromatography coupled to mass spectrometry (GC–MS) analyses were performed on a spectrometer equipped with the same column as the GC and operated under the same conditions. ^1^H-NMR, ^31^P-NMR, ^13^C-NMR, and DEPT measurements were recorded in a 300 MHz instrument using CDCl_3_ as a solvent, containing TMS as an internal standard.

The metal content of the complexes was determined by inductively coupled plasma-optical emission spectroscopy (ICP-OES, Thermo Scientific ICAP Pro) after disaggregation in aqua regia and later diluted in water before analysis. Attenuated total reflection infrared spectroscopy, performed in a JASCO FT/IR-4000, was employed to record the IR spectra (400–4000 cm^–1^) by dropping a small sample of the compound in dihcloromethane solution on the ATR crystal. Absorption ultraviolet–visible spectrophotometry (UV–vis) measurements were recorded on an Agilent Cary 60 UV–vis spectrophotometer, in 1 cm wide cuvettes and a xenon source lamp. Ultra-pressure liquid chromatography coupled to high-resolution mass spectroscopy (UPLC-HRMS) was performed using the electrospray ionization technique without previous column separation and TOF MS ES+ as a mass analyzer. Monocrystal X-ray diffraction (XRD) measurements were carried out in a Bruker D8 VENTURE PHOTON-III instrument with Mo radiation at −173 °C. Images of the Pd/C catalyst were obtained on a JEM-F2100 operated at 200 kV in dark field scanning transmission electron microscopy (DF-STEM mode), after dispersion in ethanol, supporting in a copper grid and evaporation overnight. Compounds were purified by flash column chromatography or thin-layer chromatography (TLC) under standard conditions with the appropriate mixture of solvents (typically *n*-hexane:ethyl acetate).

### Synthesis of **4a**

A suspension of sodium amide (242 mg, 5.6 mmol) in toluene (1.8 mL, 3.1 M) was slowly added for 5–10 min to a toluene solution (1.9 mL, 3 M) of indomuscone **3** (1.15 g, 5.6 mmol) in a 50 mL round bottom flask equipped with a magnetic stir bar at room temperature under continuous stirring. After 20 min, a toluene solution (1.9 mL, 3 M) of methallyl chloride (507 mg, 5.6 mmol) was added into the flask, heated in an oil bath, and continuously stirred for 12 h while the reaction mixture was refluxed. After cooling, the mixture was neutralized with HCl aqueous solution, extracted with ether, and washed with brine. The combined organic phases were dried over MgSO_4_, filtered, and concentrated under vacuum. Flash column chromatography (2% AcOEt in hexane) gave 946 mg (65% yield) of methallyl indomuscone **4a** as a yellow oil.

### Synthesis of **4b**

Indomuscone **3** (9.3 g, 45 mmol) was slowly added during 15 min to a suspension of sodium amide (2.5 g, 66 mmol, 1.5 equiv) in toluene (45 mL, 1 M) in a 100 mL round bottom flask equipped with a magnetic stir bar at room temperature under continuous stirring (750 rpm) and under a N_2_ atmosphere. Then, the mixture was heated to 45 °C in an oil bath. After 30 min at 45 °C heated with an oil bath, isobutylene oxide (4.76 g, 66 mmol, 1.5 equiv) was slowly added into the flask and the stirring continued for 6 h. After cooling, the reaction mixture was neutralized with NH_4_Cl. The aqueous phase was extracted with ethyl acetate and washed with brine. The combined organic phases were dried over MgSO_4_, filtered, and concentrated under vacuum. Flash chromatography (1% AcOEt in hexane) gave 8.8 g (70% yield) of hydroxyisobutyl indomuscone **4b** as a yellow oil.

### Synthesis of **5** from **4a**

In a 8 mL vial equipped with a magnetic stir bar and containing the catalyst (140 mg of pTsOH, 0.81 mmol, 20 mol % or 1.0 g of zeolite Beta-H), a solution of methallyl indomuscone **4a** (1.04 g, 4 mmol) in dry toluene (2 mL, 2 M) was added. The reaction mixture was open to the air, heated in an oil bath, and stirred at 70 °C for 24 h. After that time, the mixture was cooled (the zeolite filtered off if present) and neutralized with 10% aqueous sodium hydrogen carbonate solution. The aqueous phase was extracted with hexane and washed with brine. The combined organic phases were dried over MgSO_4_, filtered, and concentrated under vacuum. An aliquot was dissolved in AcOEt (1 mL) and filtered through a 20 μm nylon filter, and the resulting filtrate solution was analyzed by GC and GC–MS. The product was purified by flash column chromatography (100% hexane) to give **5** as a colorless oil (703 mg, 70%).

### Synthesis of **6**

The aromatic compound **5** (1.95 g, 7.5 mmol) was placed in a 250 mL round bottom flask equipped with a magnetic stirrer. Dichloromethane (50 mL, 0.15 M) was added into the flask. After the solid was dissolved at room temperature under magnetic stirring, Br_2_ (1.2 g, 7.5 mmol, 1 equiv) was added dropwise into the flask. After the addition of Br_2_ was completed, the resulting reaction mixture was further stirred for 15 min and then treated with Na_2_S_2_O_3_ aqueous solution (aq.), NaHCO_3_ (aq.), and brine. The organic phase was dried over Na_2_SO_4_ and filtered. Volatiles were removed from the filtrate under vacuum to give the aromatic bromide compound **6** as a yellowish solid after cooling (2.1 g, 87% yield).

### Synthesis of **2a** and **2b**

The aromatic bromide compound **6** (340 mg, 1.0 mmol) was placed in a dried 10 mL round bottom flask equipped with a magnetic stirrer, dissolved in anhydrous THF (2 mL, 0.5 M) under a nitrogen atmosphere, and cooled to −78 °C. Then, *n*-BuLi 2.5 M in hexane (0.5 mL, 1.25 mmol, 1.25 equiv) was added dropwise into the flask, and the resulting reaction mixture turned from yellow to an orange color. The reaction mixture was magnetically stirred at −78 °C for an additional 15 min before chlorodiphenylphosphine (Ph_2_PCl, 180 μL, 1.0 mmol, 1 equiv) or chlorodicyclohexylphosphine (*c*-Hex_2_PCl, 265 μL, 1.2 mmol, 1.2 equiv) was added into the flask at once. The reaction mixture was then warmed to room temperature for 90 min under stirring. The reaction mixture was then quenched with NH_4_Cl (aq.) and washed with water and brine consecutively. The organic phase was dried over Na_2_SO_4_ and filtered. The desired phosphine derivative compound product [(2,2,6,6,7,8,8-heptamethyl-3,6,7,8-tetrahydro-2*H*-indeno[4,5-*b*]furan-5-yl)diphenyl phosphane for **2a**; (2,2,6,6,7,8,8-heptamethyl-3,6,7,8-tetrahydro-2*H*-indeno[4,5-*b*]furan-5-yl) dicyclohexyl phosphane for **2b**] was purified by column chromatography and then by preparative thin-layer chromatography [TLC, 3% AcOEt in *n*-hexane; *R*_f_ (10% AcOEt in *n*-hexane) = 0.65 for **2a**; *R*_f_ (5% AcOEt in *n*-hexane) = 0.5 for **2b**] to give the desired phosphine derivative compound (colorless solid, 150 mg, 34% yield for **2a**; colorless solid, 182 mg, 40% yield for **2b**) after removing volatiles under vacuum.

### Synthesis of the Oxidized Phosphine **2b**-**oxide**

**2b** (91 mg, 0.2 mmol) was dissolved in wet THF (0.1 M) in a round bottom flask equipped with a stirring bar, heated in an oil bath, and magnetically stirred at 50 °C under 3 atm of O_2_. The mixture was monitored by GC–MS. After a 2 h reaction time, the reaction was stopped, O_2_ was removed, and the mixture was submitted to crystallization by slow evaporation of the solvent to obtain a colorless solid, 75 mg, 80% yield.

### Titration of Palladium and Phosphine **2a** by UV–Vis and ^31^P-NMR

In different round bottom flasks equipped with a stirring bars, Pd(OAc)_2_ (3 mg, 0.0133 mmol), 5 mL of MeOH (MeOD was used for ^31^P-NMR), and different amounts of phosphine (0.5–4 equiv) were added. The mixture was heated in an oil bath and allowed to stir for 10 min at 50 °C until a uniform color, without change, was achieved. This dissolution with the complex formed was measured directly by UV–vis or ^31^P NMR. The data were processed and plotted for analysis.

### Synthesis of Palladium Complexes with **2a** and **2b**

In a 100 mL round bottom flask, Pd(OAc)_2_ (29.8 mg, 0.133 mmol), phosphine **2a** (0.266 mmol, 2 equiv, 118 mg) or **2b** (121 mg), and 50 mL of the solvent (MeOH for **2a** and THF for **2b**) were added, and the mixture was heated in an aluminum plate and allowed to stir at 50 °C for 10 min until complete formation of the complex (stable orange color). Then, the mixture was concentrated in vacuo to obtain the orange solid complex (147 mg for **2a-complex** and 150 mg for **2b-complex**) to be used as a catalyst in the subsequent reactions.

### Typical Procedure for the Telomerization Reaction

Isoprene **7** (68.1 mg, 1 mmol), 0.05 mmol of NaOMe, palladium complex (11.0 mg, 0.01 mmol) with **2a** (11.4 mg, 1 mol %), and MeOH (250 μL, 4 M) were introduced into a 2 mL glass vial, which was then sealed. The mixture was stirred for 1 h at 60 °C heated in an aluminum plate, taking 5 μL aliquots for analysis by GC (the aliquot was diluted in DCM with *n*-dodecane as an external standard to monitor the reaction process). Once finished, 1 mL of water was added, and the mixture was extracted with 2 mL of ethyl acetate (three times), washed with brine, dried over MgSO_4_, and filtered into a 50 mL flask and volatiles were removed in vacuo. To purify the complex, flash chromatography on silica (with a mixture 1:100, EtOAc:hexane) was performed, obtaining 165 mg of the corresponding ethers **8** with a yield of 98% as a yellow oil.

### Typical Procedure for the Buchwald–Hartwig Reaction

Aryl chloride (0.25 mmol), amine (0.5 mmol), NaO^*t*^Bu (36 mg, 0.374 mmol), Pd(OAc)_2_ (3.6 mg, 2.5 mol %), and phosphine **2b** (7.7 mg, 7.5 mol %) were mixed in dioxane (2.0 mL, 0.13 M). The mixture was stirred at 80 °C heated in an aluminum plate in a sealed vial and monitored by GC/GC–MS. When the reaction ends, water was added and the mixture was extracted with Et_2_O (three times). The combined organic layers were washed with H_2_O and brine, dried over anhydrous MgSO_4_, filtered, and concentrated in vacuo. The residue was purified by column chromatography with a gradient eluent of 5–30% ethyl acetate in hexanes to give the desired product.

### Typical Procedure for the Suzuki Cross-Coupling Reaction

Aryl chloride (0.12 mmol), boronic acid (0.19 mmol), Cs_2_CO_3_ (81.45 mg, 0.25 mmol), Pd(OAc)_2_ (1.7 mg, 3 mol %), and phosphine **2b** (3.4 mg, 5 mol %) were mixed in dioxane (0.4 mL, 0.3 M). The mixture was stirred at 80 °C heated in an aluminum plate in an ambient atmosphere and monitored by GC/GC–MS. When the reaction ends, water was added and the mixture was extracted with Et_2_O (three times). The combined organic layers were washed with H_2_O and brine, dried over anhydrous MgSO_4_, filtered, and concentrated in vacuo. The residue was purified by column chromatography with a gradient eluent of 5% ethyl acetate in hexanes to give the desired product.

### Typical Procedure for the Semi-Hydrogenation Reaction of **18**

All the semi-hydrogenation reactions were performed in batch in a 6 mL round bottom vial, with 0.5 mL of reaction volume and a stirring magnet. The solvent was ethanol, at a 0.3 M concentration when the pressure was set at 1 bar of H_2_, and at a 0.5 M concentration under higher pressures of H_2_. The reactions were conducted at 25 °C heated in an aluminum plate and stirred at 450 rpm. The H_2_ pressure was kept constant by refilling the reactor periodically, so enough H_2_ was always present to fully hydrogenate the alkyne to the corresponding alkane. Yields were obtained by GC, and GC-coupled mass spectrometry and NMR were used to identify the products, besides comparison with pure product samples.

## Data Availability

The data underlying this study are available in the published article and its Supporting Information.

## Supporting Information Available

Additional experimental data, Figures S1–S8, Tables S1–S7, compound characterization, NMR and FT-IR copies, and summary of crystallographic data (PDF)
