# Supplementary material for: Indomuscone-Based Sterically Encumbered Phosphines as Ligands for Palladium-Catalyzed Reactions
Source: J Org Chem. 2023 Apr 7;88(9):5962–71. doi: 10.1021/acs.joc.3c00314 (PMC10167692; doi:10.1021/acs.joc.3c00314)
Supplement: Supplementary file 1 — jo3c00314_si_001.pdf [file jo3c00314_si_001.pdf]

SUPPORTING INFORMATION

Indomuscone-based sterically-encumbered phosphines as ligands for palladium-catalyzed reactions

Francisco Garnes-Portolés,[a] Sergio Sanz–Navarro,[a] Jordi Ballesteros–Soberanas,[a] Ana Collado–Pérez,[b] Jorge Sánchez–Quesada,[b] Estela Espinós–Ferri[b] and Antonio Leyva–Pérez.[a,*]

[a] Instituto de Tecnología Química. Universitat Politècnica de València–Consejo Superior de Investigaciones Científicas. Avda. de los Naranjos s/n, 46022, Valencia, Spain.
[b] International Flavours & Fragrances Inc., Avda Felipe Klein 2, 12580, Benicarló, Castellón, Spain.
Corresponding author's email: anleyva@itq.upv.es

<u>Table of contents</u>

Supporting Figures (Figures S1-S8) p. S2-S7

Supporting Tables (Table S1-S7) p. S8-S14

Compound characterization p. S15-S20

NMR copies p. S21-S32

FT-IR copies p. S33-S35

Summary of crystallographic data p. S36

References p. S37

Supporting Figures

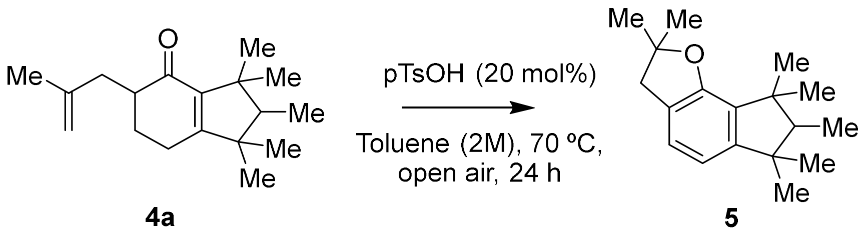

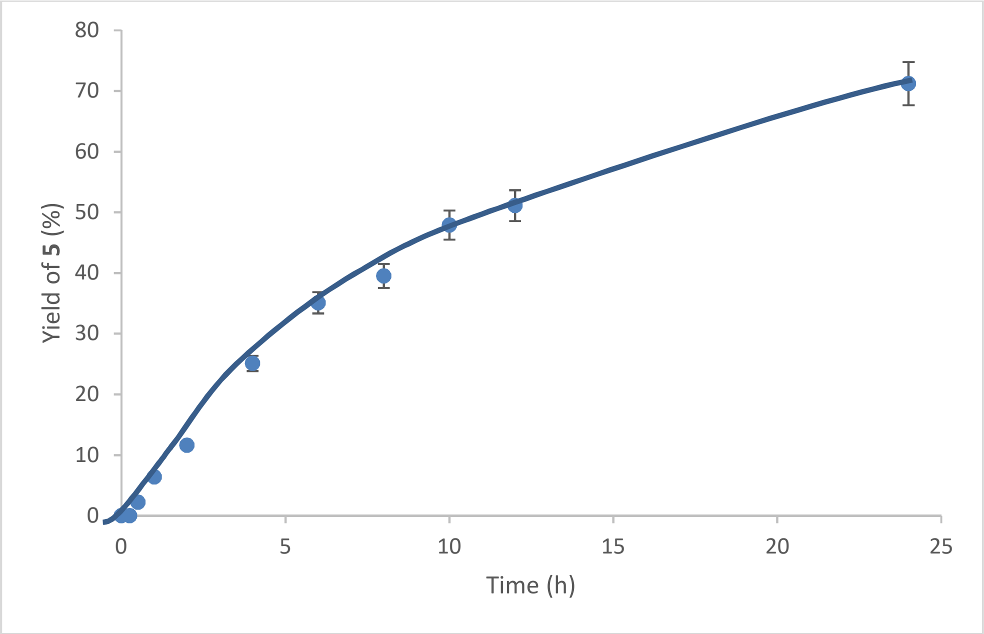

Figure S1. Kinetic plots for the cyclization of methallyl indomuscone **4a** with pTsOH (20 mol%). For numeric results see Table S3. Error bars account for a 5% uncertainty.

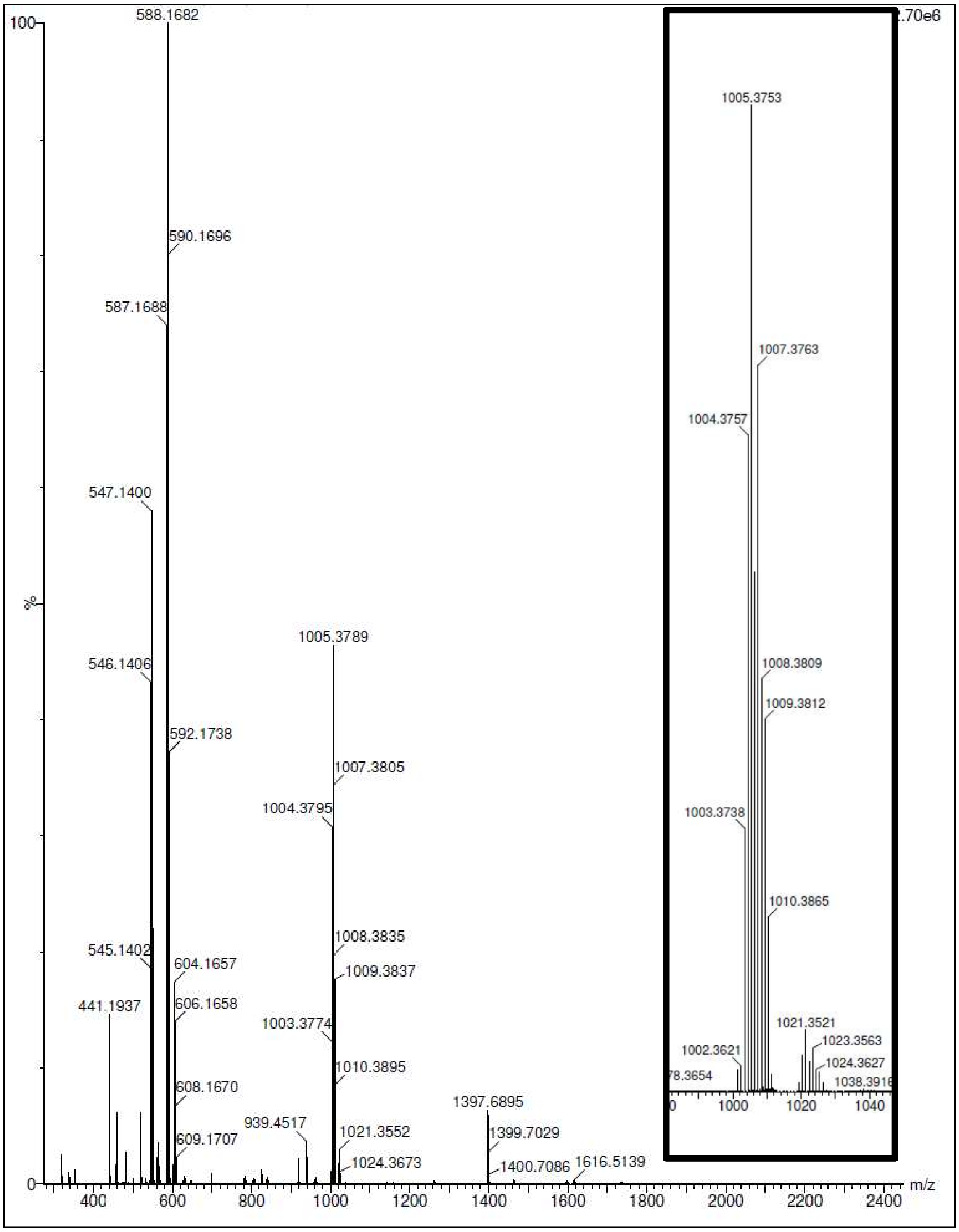

Figure S2. Ultra-pressure liquid chromatography coupled to high resolution mass spectroscopy (UPLC-HRMS) results for complex Pd(**2a**)$_2$OAc.

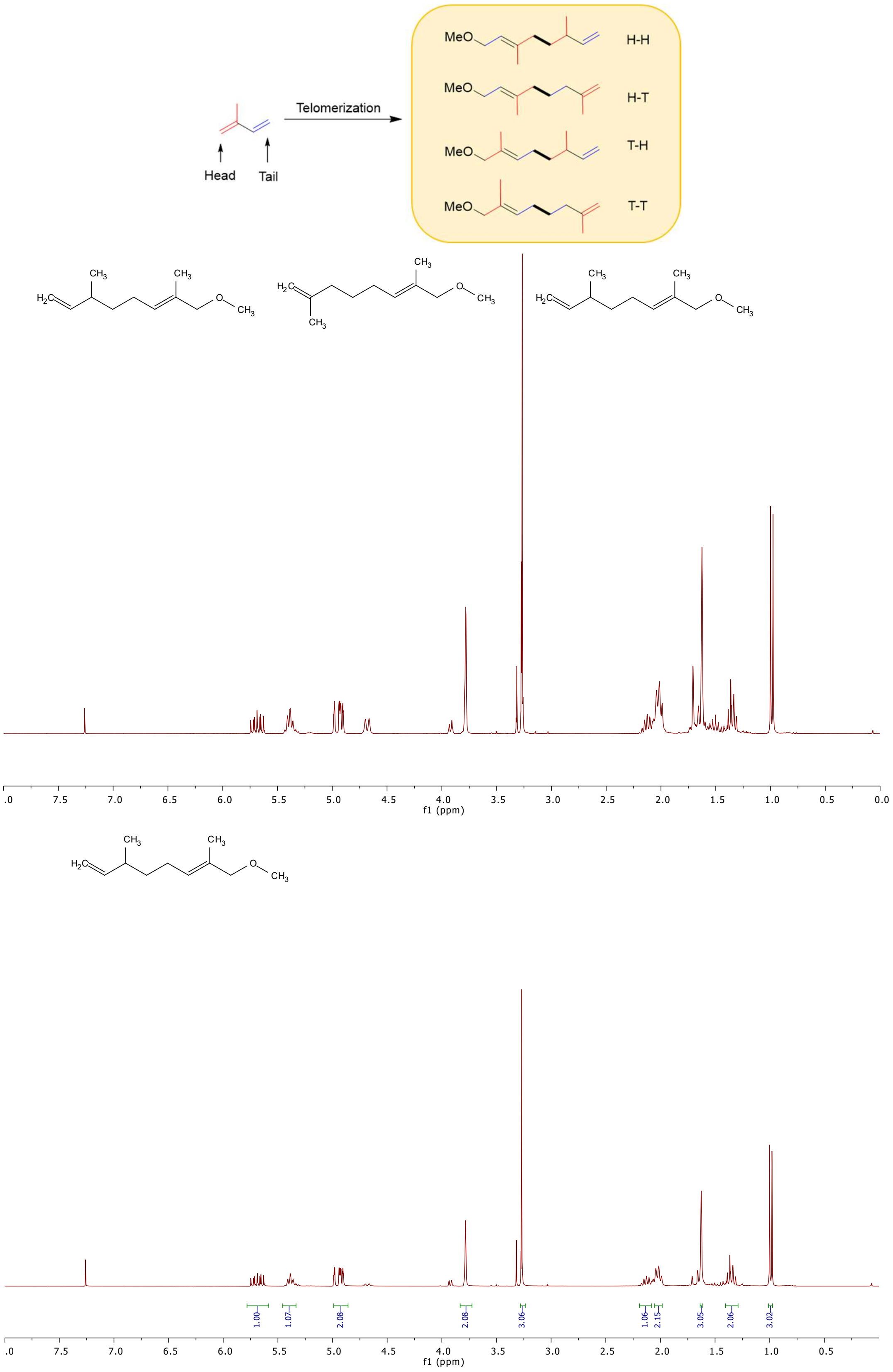

Figure S3. Top: Different potential products during the telomerization of isoprene **7** catalyzed by palladium phosphine complexes. Bottom: ^{1}H-NMR spectra of the reaction with either phosphine **1a** (up) or **2a** (down, entries 3 and 4 in Table 1 of the main text, respectively).

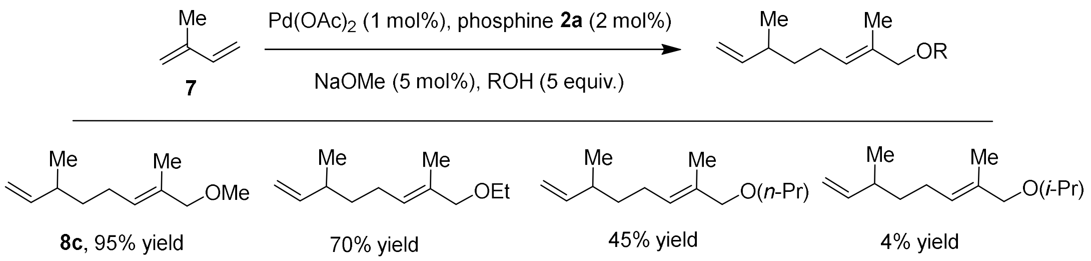

Figure S4. Catalytic results for the telomerization reaction of isoprene **7** and different alcohols catalyzed by the 1:2 $Pd(OAc)_2$-phosphine **2a** complex catalyst under optimized reaction conditions. GC results.

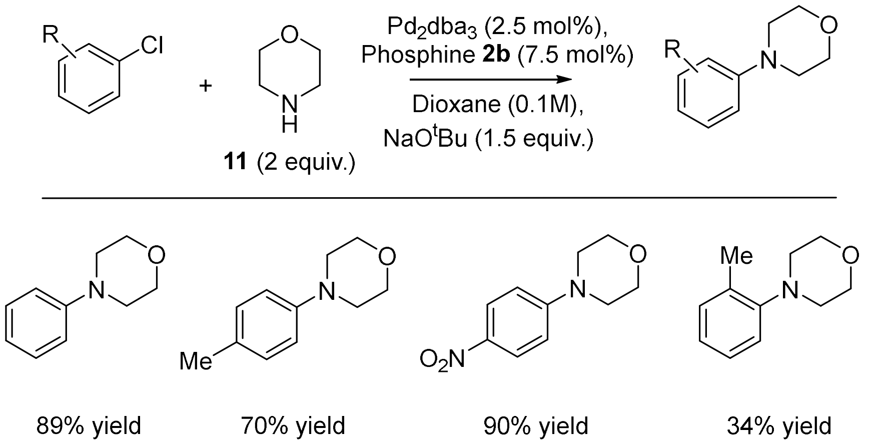

Figure S5. Catalytic results for the coupling between chloroderivatives and morpholine **11** under optimized reaction conditions, with $Pd_2(dba)_3$ and phosphine ligand **2b** as catalysts. GC results.

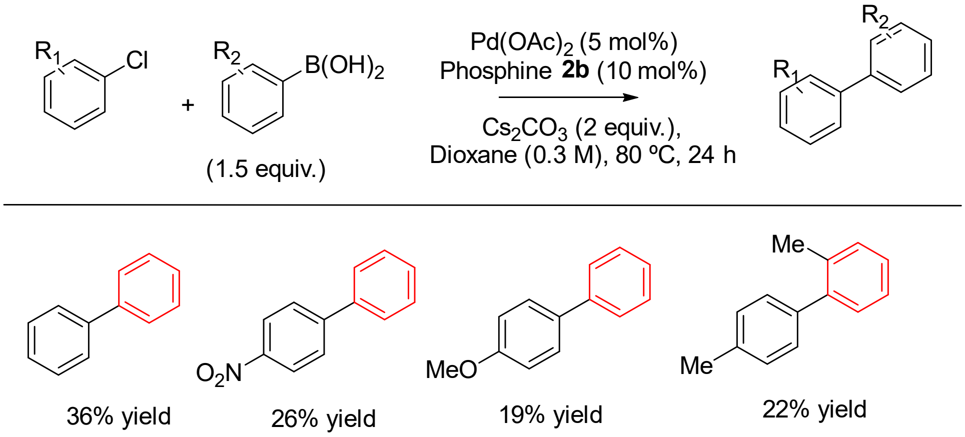

Figure S6. Catalytic results for the cross-coupling reaction between different chloroaromatics and arylboronic acids under optimized reaction conditions, catalyzed by the 1:2 $Pd(OAc)_2$-phosphine **2b** complex catalyst. GC results.

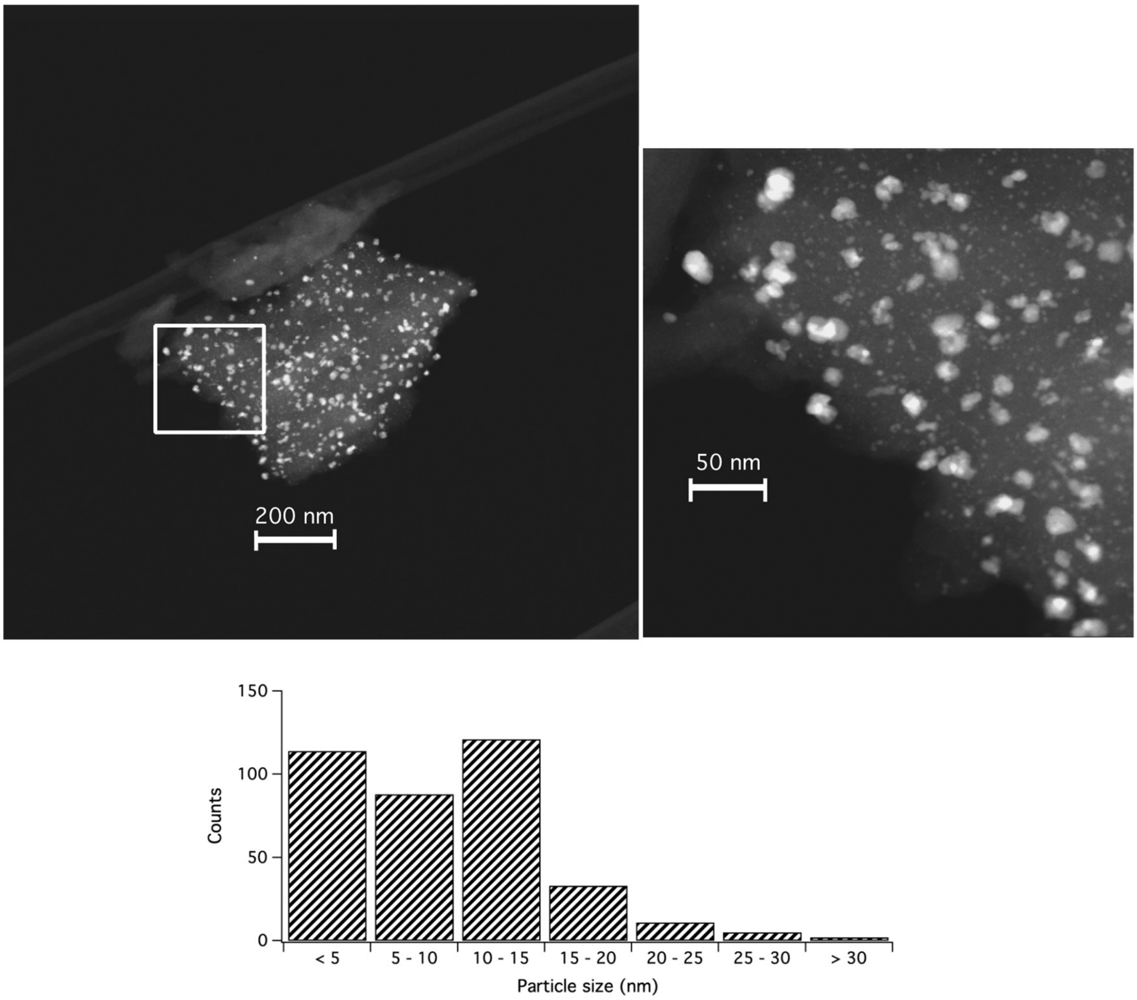

Figure S7. Dark field STEM images of a representative commercial Pd on carbon sample (1 wt%) from Merck-Millipore Sigma.

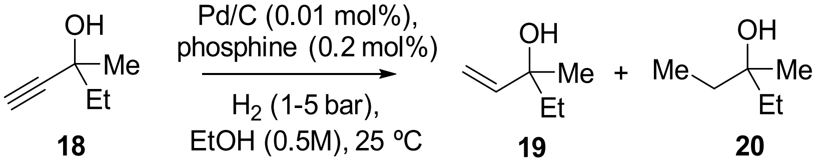

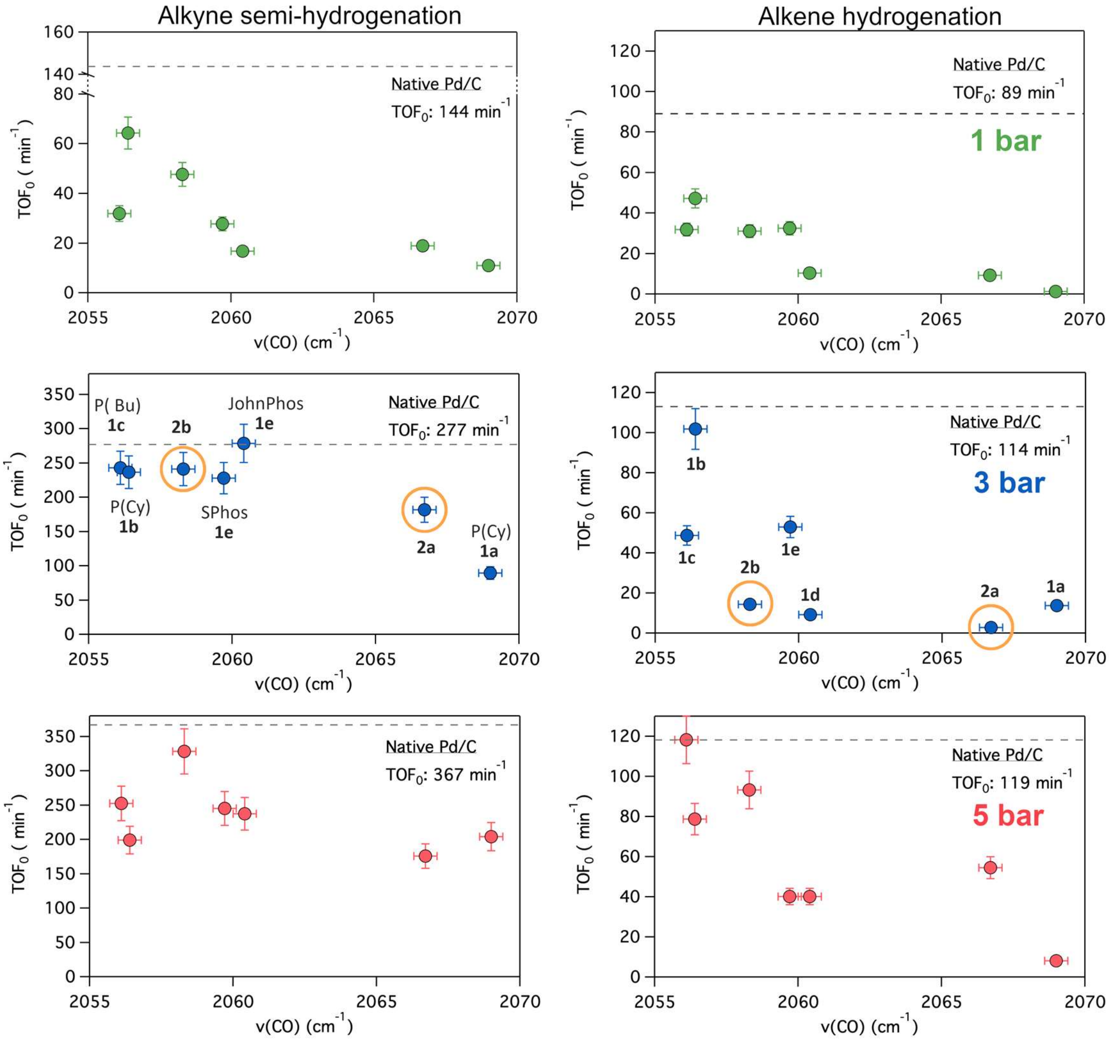

Figure S8. Correlations between turnover frequencies in the alkyne to alkene (left) and alkene to alkane (right) hydrogenation reactions and the phosphine electronic properties, expressed in terms of ν(CO, cm^{-1}) of the corresponding $Ni(CO)_3(PR_3)$ complex, at different H_2 reaction pressures. The reactions were performed with Pd/C (1 wt%, 0.01 mol%) at 1 bar (top, green), 3 bar (middle, blue) and 5 bar (bottom, red) and 1:20 ratio Pd : phosphine. Vertical error bars represent ± 10% uncertainty, horizontal error bars represent the ± 0.3 cm^{-1} of uncertainty. The phosphines synthesized in this work (**2a**, **2b**) are encircled.

Supporting Tables

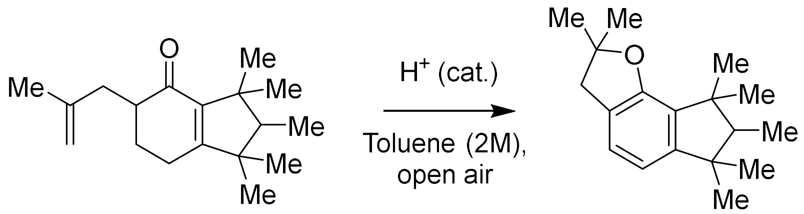

4a **5**

Table S1. Catalytic results for the cyclization of methallyl indomuscone **4a** with homogeneous acid and zeolite catalysts.

**Entry**	**Acid Catalyst (amount relative to 4a)**	**T (ºC)**	**t (h)**	**Conv (%)**	**Yield (%)**
1	MSA (20 mol%)	25	18	100	25
2	**pTsOH (20 mol%)**			90	20
3		25	48	**100**	20
4		50	36		45
**5**		**70**	**24**		**70**
**6**	**H-Beta (CP–811, 100 wt%)**	**110**	**12**	**100**	**70**
7	H–USY (CBV–720, 100 wt%)	110	12	100	50

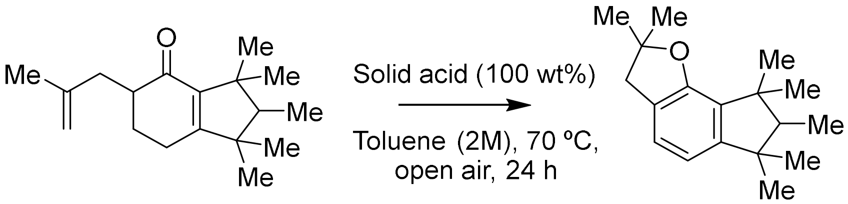

4a **5**

Table S2. Catalytic results for the cyclization of methallyl indomuscone **4a** with other solid acid catalysts.

<table>
<tr><th>Entry</th><th>Acid Catalyst</th><th>Conv (%)</th><th>Yield (%)</th></tr>
<tr><td>1</td><td>Zeolite H-Beta (CP–811)</td><td>55</td><td>25</td></tr>
<tr><td>2</td><td>Zeolite H–USY (CBV–720)</td><td>40</td><td>10</td></tr>
<tr><td>3</td><td>Zeolite H–USY (CBV–760)</td><td>40</td><td>10</td></tr>
<tr><td>4</td><td>Zeolite H–USY (CBV–780)</td><td>30</td><td>10</td></tr>
<tr><td>5</td><td>Zeolite H–USY (CBV–400)</td><td>50</td><td>15</td></tr>
<tr><td>6</td><td>Sulfonic Acid Resin Amberlyst 15</td><td>40</td><td>5</td></tr>
<tr><td>7</td><td>Sulfonic Acid Resin Amberlyst 16</td><td>45</td><td>5</td></tr>
<tr><td>8</td><td>Sulfonic Acid Resin Amberlyst 20</td><td>45</td><td>5</td></tr>
<tr><td>9</td><td>Zeolite NaX</td><td rowspan="2">0</td><td rowspan="2">0</td></tr>
<tr><td>10</td><td>Zeolite NaY</td></tr>
</table>

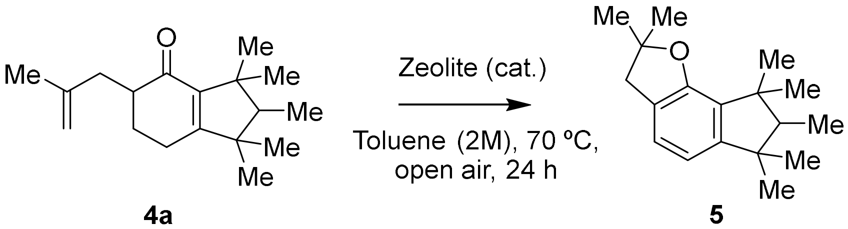

4a **5**

Table S3. Catalytic results for the cyclization of methallyl indomuscone **4a** with different amounts of zeolite H-Beta and H-USY catalysts.

Entry	Zeolite Catalyst	Amount (wt%)	Conv (%)	Yield (%)
1	H-Beta	100	55	25
2		50	30	5
3		25	30	5
4		10	20	5
5	H-USY	100	40	10
6		50	35	5
7		25	20	5
8		10	15	0

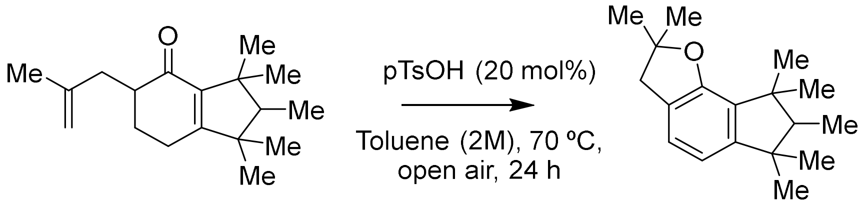

4a **5**

Table S4. Catalytic results for the cyclization of methallyl indomuscone **4a** with pTsOH (20 mol%) over time.

Entry	Reaction Time (hrs)	Yield (%)
1	0.25	<1
2	0.5	2
3	1	7
4	2	11
5	4.5	24
6	6.5	33
7	8	38
8	11.5	48
9	13.5	51
10	24	70

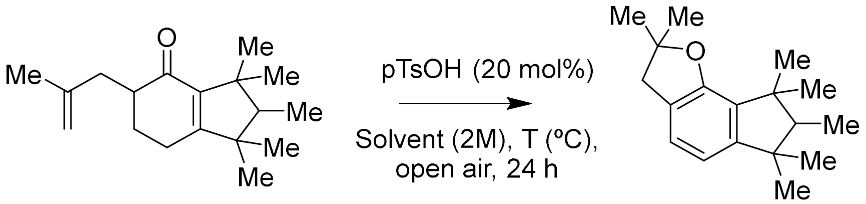

4a **5**

Table S5. Catalytic results for the cyclization of methallyl indomuscone **4a** with pTsOH (20 mol%) in different solvents.

**Entry**	**Solvent**	**T (ºC)**	**Conv (%)**	**Yield (%)**
1	Toluene	70	100	70
2	THF	50	90	45
3	Dioxane	70	90	55
4	Acetonitrile	60	20	0
5	Ethanol	60	80	50
6	H_2O	70	10	0

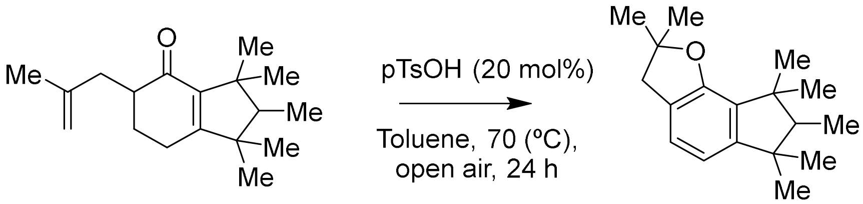

4a **5**

Table S6. Catalytic results for the cyclization of methallyl indomuscone **4a** with pTsOH (20 mol%) in toluene at different concentrations.

Entry	Concentration (M)	Conv (%)	Yield (%)
1	0.1	80	50
2	0.25	90	50
3	1	100	40
4	2	100	70
5	3	100	40

Table S7. Electronic properties, expressed as the carbonyl vibrational frequency of the corresponding $Ni(CO)_3(PR_3)$ complex, and the cone angle formed by the phosphine substituents (Tolman cone angle) in the phosphines used in this work.

**Type**	**Number**	**Phosphine**	**$Ni(CO)_3L$ $\upsilon(CO)$, cm^{-1}**	**Tolman Cone Angle[a]**
PAr_3	**1a**	PPh_3	2069.0 ± 0.3	145.0
$PAlk_3$	**1b**	PCy_3	2056.4 ± 0.3	170.0
	**1c**	$P(^tBu)_3$	2056.1 ± 0.3	182.0
Buchwald	**1d**	JohnPhos	2060.4[b] ± 0.3	184.1[c]
	**1e**	SPhos	2059.7[d] ± 0.3	204.4[c]
Fragrance-based (this work)	**2a**	PPh_2(indomuscone derivative)	2066.7[e] ± 1.5	195.0[f]
	**2b**	P*c*-Hex_2(indomuscone derivative)	2058.3[e] ± 1.5	205.0[f]

a) From Ref. S1
b) Estimated as Ph
c) From Ref. S2
d) Estimated as (o-C_6H_4OMe)
e) Given estimated range ± 1.5
f) Calculated in this work

Compound characterization.

- *1,1,2,3,3-Pentamethyl-5-(2-methylallyl)-1,2,3,5,6,7-hexahydro-4H-inden-4-one* (**4a**):

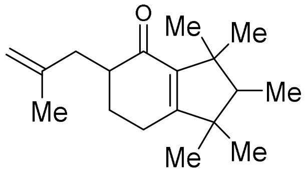

^{1}H – NMR ($CDCl_3$, 300 MHz) δ (ppm)

^{1}H NMR (300 MHz, Chloroform-*d*) δ 4.73 (d, *J* = 25.9 Hz, 2H), 2.62 (dd, *J* = 14.0, 4.4 Hz, 1H), 2.31 (tq, *J* = 9.5, 4.9 Hz, 2H), 2.18 (dd, *J* = 8.4, 5.0 Hz, 1H), 2.13 – 1.91 (m, 2H), 1.71 (s, 3H), 1.69 – 1.52 (m, 3H), 1.20 (s, 3H), 1.05 (s, 3H), 1.01 (s, 3H), 0.92 (s, 3H), 0.87 (d, *J* = 7.5 Hz, 3H).

^{13}C – NMR ($CDCl_3$, 75 MHz) δ (ppm)

^{13}C{1H} NMR (75 MHz, Chloroform-*d*) δ 199.7, 169.1, 143.8, 141.2, 112.2, 53.2, 47.4, 45.33, 45.1, 37.9, 28.0, 27.2, 26.6, 21.9, 21.7, 21.6, 21.1, 7.8.

GC – MS and HR – MS:

Calculated for $C_{18}H_{28}O$: 260.21, found: 260.2.

HRMS (ESI) m/z: [M+H$^+$] calculated for $C_{18}H_{29}O$: 261.2220, found: 261.2218.

FT – IR:

2951.5 (i), 1659.4 (vi), 1448.3 (m), 1384.6 (m).

- *5-(2-Hydroxy-2-methylpropyl)-1,1,2,3,3-pentamethyl-1,2,3,5,6,7-hexahydro -4H-inden-4-one* (**4b**):

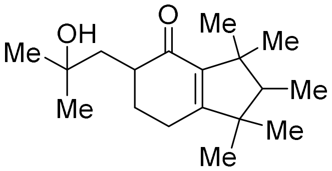

^{1}H – NMR ($CDCl_3$, 300 MHz) δ (ppm)

^{1}H NMR (300 MHz, Chloroform-*d*) δ 4.27 (s, 1H), 2.57 – 2.38 (m, 1H), 2.35 – 2.15 (m, 2H), 2.13 – 1.93 (m, 2H), 1.84 – 1.66 (m, 2H), 1.64 – 1.49 (m, 1H), 1.22 (s, 3H), 1.16 (d, *J* = 2.7 Hz, 3H), 1.13 (s, 3H), 1.00 (d, *J* = 10.7 Hz, 3H), 0.95 (d, *J* = 4.3 Hz, 3H), 0.88 (d, *J* = 3.3 Hz, 3H), 0.82 (d, *J* = 7.4 Hz, 3H).

^{13}C – NMR (CDCl$_3$, 75 MHz) δ (ppm)

^{13}C{1H} NMR (75 MHz, Chloroform-*d*) δ 202.5, 170.8, 141.6, 69.0, 53.3, 47.3, 45.3, 44.4, 43.9, 32.6, 31.3, 28.2, 27.1, 26.3, 22.9, 22.1, 21.2, 7.7.

GC – MS

Calculated for $C_{18}H_{30}O_2$: 278.22, found: 261.4 (-OH).

- *2,2,6,6,7,8,8-Heptamethyl-3,6,7,8-tetrahydro-2H-indeno[4,5-b]furan* (**5**):

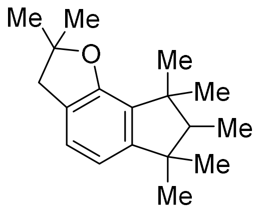

^{1}H – NMR (CDCl$_3$, 300 MHz) δ (ppm)

^{1}H NMR (300 MHz, Chloroform-*d*) δ 7.01 (d, *J* = 7.5 Hz, 1H), 6.68 (d, *J* = 7.5 Hz, 1H), 2.97 (s, 2H), 2.03 – 1.84 (m, 1H), 1.49 (s, 6H), 1.41 (s, 3H), 1.30 (s, 3H), 1.17 (s, 3H), 1.09 (s, 3H), 1.01 (d, *J* = 7.4 Hz, 3H).

^{13}C – NMR (CDCl$_3$, 75 MHz) δ (ppm)

^{13}C{1H} NMR (75 MHz, Chloroform-*d*) δ 155.1, 152.4, 132.0, 125.3, 123.2, 113.6, 86.4, 54.7, 45.4, 44.3, 42.5, 29.1, 28.3 (d, *J* = 1.5 Hz), 27.9, 26.3, 22.7, 8.3.

GC – MS and HR – MS:

Calculated for $C_{18}H_{26}O$: 258.2, found: 258.1.

HRMS (ESI) m/z: [M+H$^+$] calculated for $C_{18}H_{27}O$: 259.2064, found: 259.2055.

FT – IR:

2967.9 (i), 1593.8 (w), 1437.7 (vi), 1273.7 (i).

- *5-Bromo-2,2,6,6,7,8,8-heptamethyl-3,6,7,8-tetrahydro-2H-indeno[4,5-b] furan* (**6**):

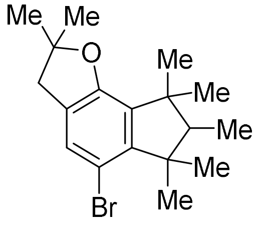

^{1}H – NMR ($CDCl_3$, 401 MHz) δ (ppm)

^{1}H NMR (401 MHz, Chloroform-*d*) δ 7.01 (t, *J* = 2.3 Hz, 1H), 2.81 (d, *J* = 1.2 Hz, 2H), 1.76 (q, *J* = 7.4 Hz, 1H), 1.38 (s, 3H), 1.36 (s, 3H), 1.34 (s, 3H), 1.27 (s, 3H), 1.09 (s, 3H), 1.00 (s, 3H), 0.87 (d, *J* = 7.4 Hz, 3H).

^{13}C – NMR ($CDCl_3$, 101 MHz) δ (ppm)

^{13}C{1H} NMR (101 MHz, Chloroform-*d*) δ 154.8, 147.8, 135.1, 128.1, 128.1, 108.4, 87.1, 55.2, 48.0, 44.1, 42.0, 28.2, 28.1, 27.8, 27.6, 22.5, 21.8, 7.9.

GC – MS and HR – MS:

Calculated for $C_{18}H_{25}BrO$: 336.11, found: 336.1.

HRMS (ESI) m/z: [M-H$^+$] calculated for $C_{18}H_{24}BrO$: 335.1009, found: 335.1012.

FT – IR:

2962.1 (m), 1737.5 (i), 1442.5 (vi), 1270.9 (vi), 1238.1 (vi).

- *(2,2,6,6,7,8,8-Heptamethyl-3,6,7,8-tetrahydro-2H-indeno[4,5-b]furan-5-yl) diphenylphosphane* (**2a**):

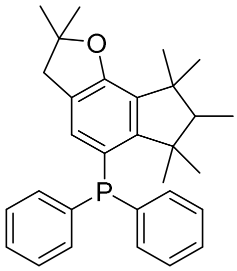

^{1}H – NMR ($CDCl_3$, 401 MHz) δ (ppm)

^{1}H NMR (401 MHz, Chloroform-*d*) δ 7.42 – 7.16 (m, 10H), 6.71 (dd, *J* = 4.5, 1.2 Hz, 1H), 2.80 (dd, *J* = 6.3, 1.1 Hz, 2H), 1.53 (s, 3H), 1.45 (s, 3H), 1.43 (s, 3H), 1.39 (s, 3H), 1.38 (s, 3H), 1.12 (s, 3H), 0.96 (d, *J* = 7.3 Hz, 3H).

^{13}C – NMR ($CDCl_3$, 101 MHz) δ (ppm)

^{13}C{1H} NMR (101 MHz, Chloroform-*d*) δ 157.7, 157.4, 156.6, 139.4 (dd, *J* = 71.8, 11.7 Hz), 133.4 (dd, *J* = 19.2, 2.5 Hz), 132.6 (d, *J* = 9.7 Hz), 131.3 (d, *J* = 2.2 Hz), 128.2 (d, *J* = 3.6 Hz), 128.2 (d, *J* = 3.1 Hz), 127.8 (d, *J* = 15.2 Hz), 87.0, 55.2, 48.1, 43.7, 42.1, 30.9 (d, *J* = 19.7 Hz), 28.3 (d, *J* = 8.0 Hz), 27.8, 24.6 (d, *J* = 7.9 Hz), 22.8, 14.1, 8.0.

GC – MS, ^{31}P – NMR and HR – MS

Calculated for $C_{30}H_{35}OP$: 442.24, found: 442.3. // ^{31}P NMR (162 MHz, Chloroform-*d*) δ -18.29.

HRMS (ESI) m/z: [M-H$^+$] calculated for $C_{30}H_{34}OP$: 441.2347, found: 441.2343

FT – IR:

2967.9 (w), 1572.7 (w), 1431.9 (i), 1277.6 (m), 1105.0 (m).

- *Dicyclohexyl(2,2,6,6,7,8,8-heptamethyl-3,6,7,8-tetrahydro-2H-indeno[4,5-b]furan-5-yl)phosphane* (**2b**):

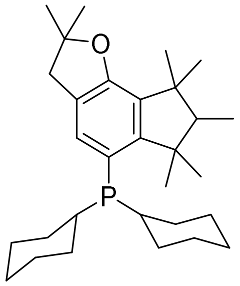

^{1}H – NMR ($CDCl_3$, 401 MHz) δ (ppm)

^{1}H NMR (401 MHz, Chloroform-*d*) δ 7.03 (d, *J* = 3.0 Hz, 1H), 2.86 (d, *J* = 1.1 Hz, 2H), 1.93 – 1.82 (m, 2H), 1.76 – 1.65 (m, 7H), 1.51 (s, 3H), 1.49 (s, 3H), 1.40 (s, 3H), 1.38 (s, 3H), 1.29 (s, 3H), 1.27 – 1.21 (m, 4H), 1.18 (s, 3H), 1.14 – 1.03 (m, 7H), 0.99 (s, 3H), 0.85 (d, *J* = 7.3 Hz, 3H).

^{13}C – NMR ($CDCl_3$, 101 MHz) δ (ppm)

^{13}C{1H} NMR (101 MHz, Chloroform-*d*) δ 158.1, 156.0, 128.4, 125.4, 121.9, 121.7, 86.6, 55.4, 48.0, 43.3, 42.2, 36.6, 31.6, 31.3, 30.3, 28.4, 27.8, 26.5, 22.9, 22.7, 14.1, 8.1.

GC – MS, HR – MS and ^{31}P – NMR:

Calculated for $C_{30}H_{47}OP$: 454.34, found: 454.3.

HRMS (ESI) m/z: [M+H$^+$] calculated for $C_{30}H_{48}OP$: 455.3445, found: 455.3424.

^{31}P NMR (162 MHz, Chloroform-*d*) δ -16.23.

FT – IR:

2914.9 (vi), 1570.7 (w), 1435.7 (vi), 1275.7 (i), 1117.5 (m).

➤ *Bis(2,2,6,6,7,8,8-heptamethyl-3,6,7,8-tetrahydro-2H-indeno[4,5-b]furan-5-yl) diphenylphosphane bisacetate palladium(II) complex [Pd(**2a**)$_2$(OAc)$_2$]*:

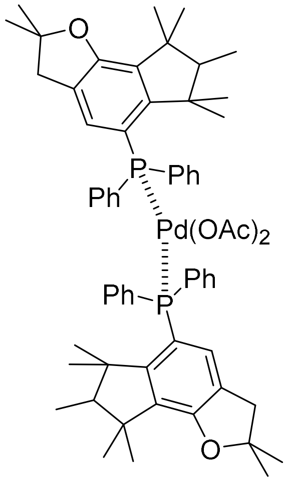

**^{1}H – NMR ($CDCl_3$, 401 MHz) δ (ppm)**

^{1}H NMR (401 MHz, Chloroform-*d*) δ 7.65 – 7.31 (m, 20H), 6.65 (s, 1H), 6.61 (s, 1H), 2.80 (dd, *J* = 9.0, 1.2 Hz, 4H), 1.93 – 1.79 (s, 6H), 1.71 (q, *J* = 7.3 Hz, 2H), 1.47 (s, 6H), 1.45 (s, 6H), 1.42 (s, 6H), 1.35 (s, 6H), 1.19 (s, 6H), 1.14 (s, 6H), 0.91 (d, *J* = 7.3 Hz, 6H).

**^{13}C – NMR ($CDCl_3$, 101 MHz) δ (ppm)**

^{13}C{1H} NMR (101 MHz, Chloroform-*d*) δ 158.7, 158.7, 158.4, 136.5 (d, *J* = 43.4 Hz), 134.7 (d, *J* = 11.8 Hz), 132.1 (dd, *J* = 34.8, 9.4 Hz), 131.1 (d, *J* = 10.1 Hz), 130.5 (d, *J* = 16.3 Hz), 128.2 (dd, *J* = 11.8, 2.3 Hz), 124.8, 118.0, 87.7, 55.0, 48.8, 43.7, 42.0, 28.3, 28.2, 28.0, 24.2, 22.2, 7.9.

**HRMS and ^{31}P– NMR:**

HRMS (ESI) m/z: [M-OAc,-3x Me] Calculated for $C_{18}H_{16}O_3$: 1108.4152, found: 1004.3757.

^{31}P NMR (162 MHz, Chloroform-*d*) δ 33.90.

**FT – IR:**

2967.9 (w), 2359.5 (w), 1572.7 (m), 1435.7 (i), 1097.3 (m).

➤ *(E)-8-methoxy-3,7-dimethylocta-1,6-diene (**8c**):*[S3]

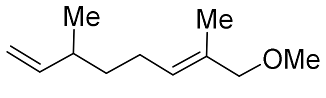

GC – MS:

Calculated for $C_{11}H_{20}O$: 168.2, found: 168.1.

<u>**NMR copies.**</u>

^{1}H NMR (300 MHz, Chloroform-*d*) **(4a)**

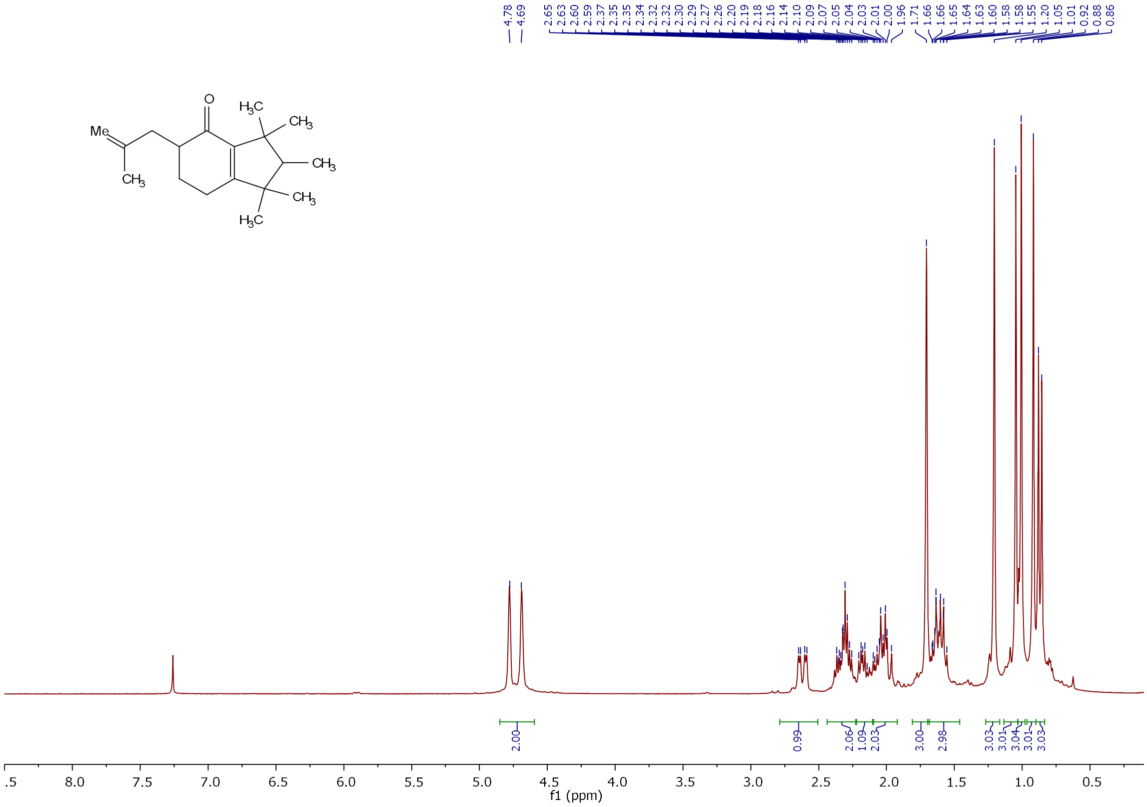

^{13}C{1H}(75 MHz, Chloroform-*d*) **NMR (4a)**

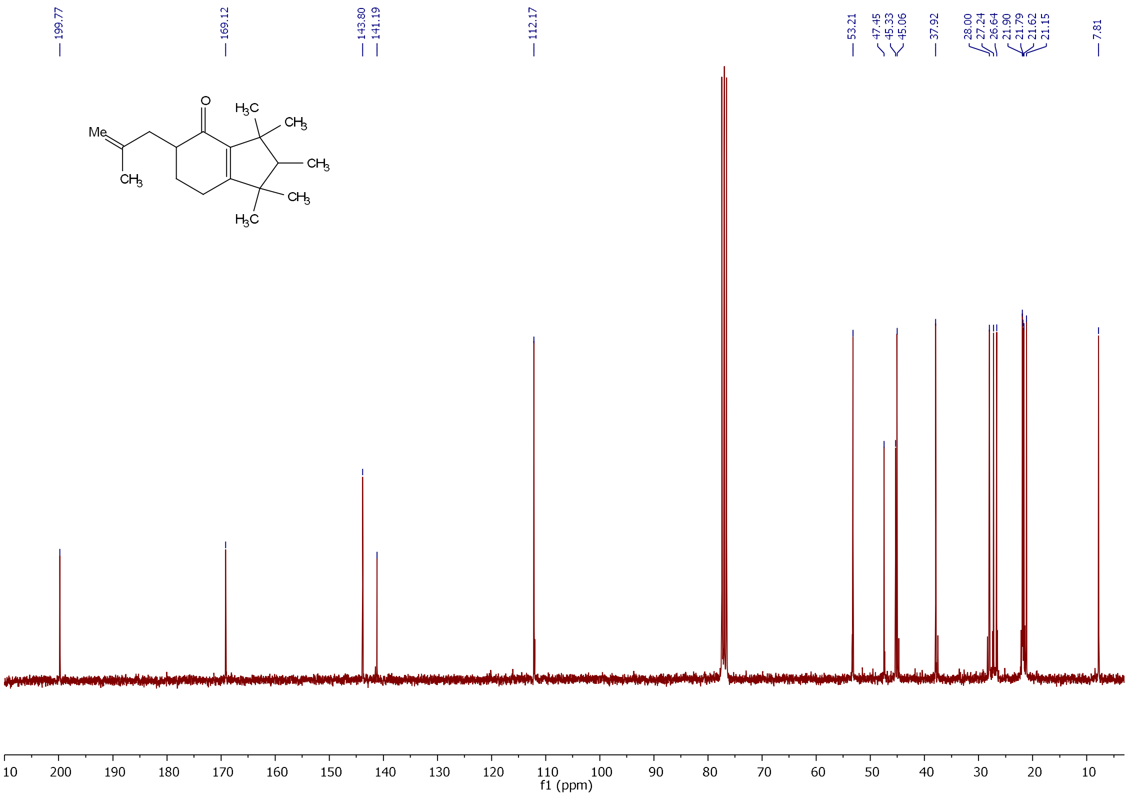

DEPT(75 MHz, Chloroform-*d*) **(4a)**

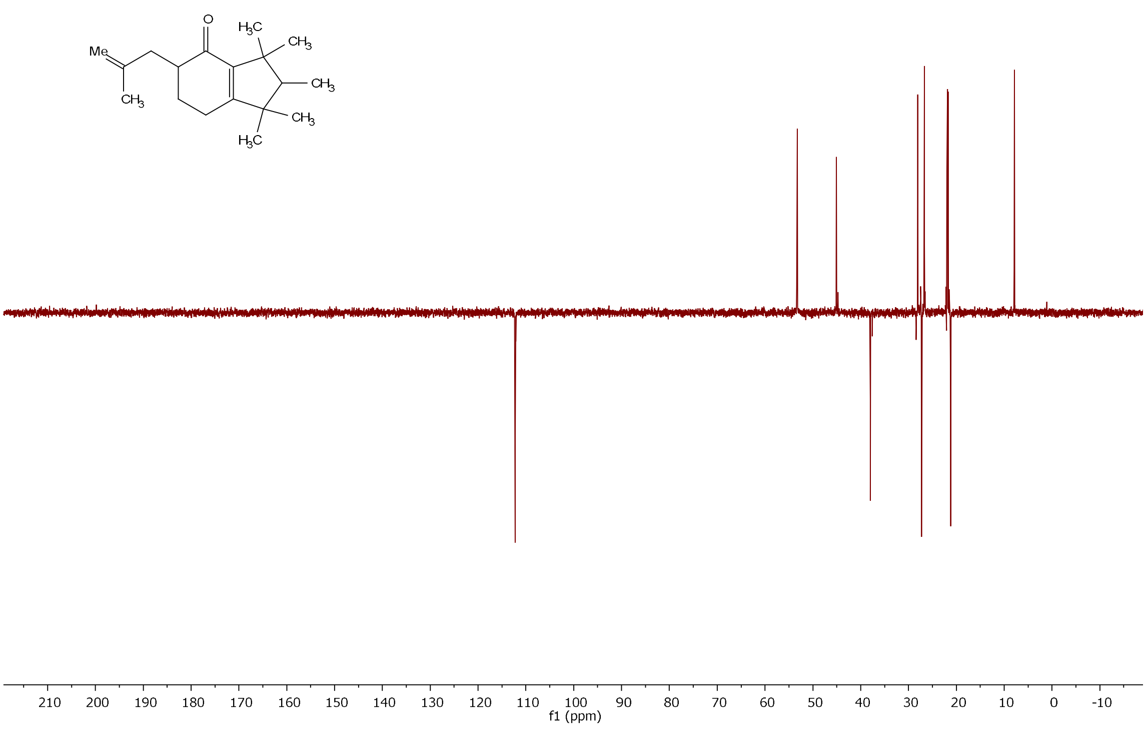

^{1}H (300 MHz, Chloroform-*d*) **NMR (4b)**

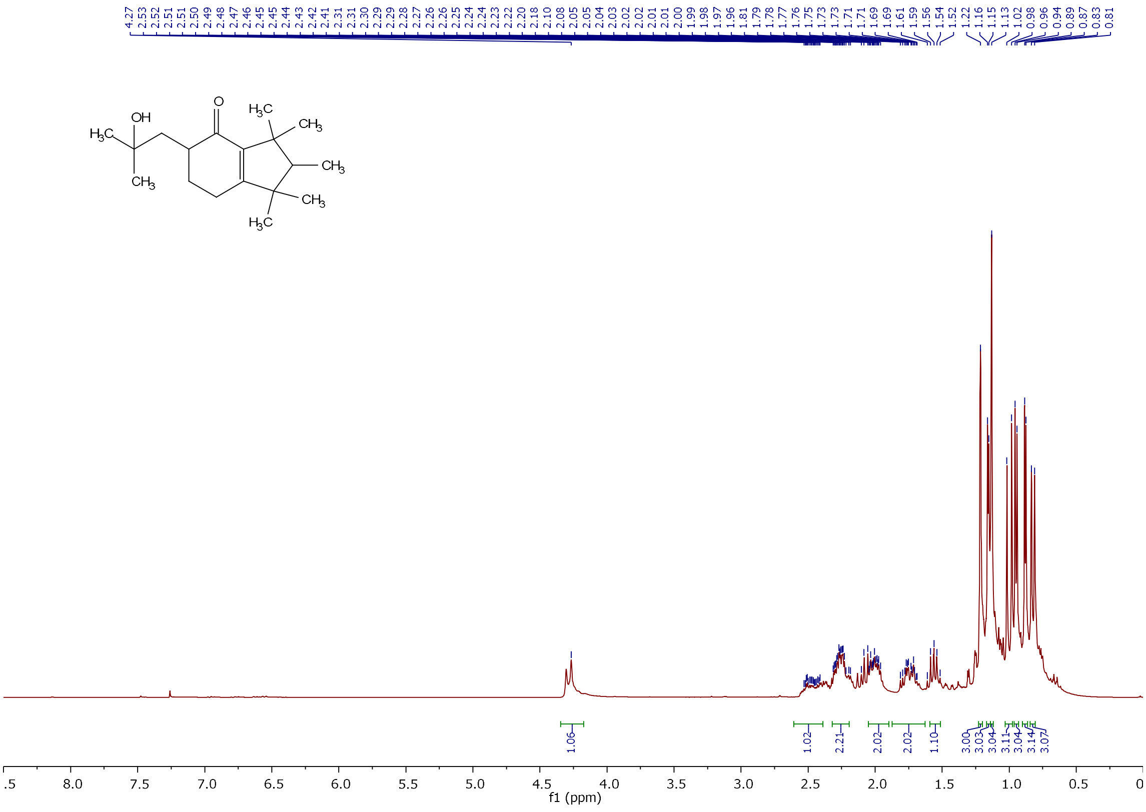

^{13}C{1H}(75 MHz, Chloroform-*d*) **NMR (4b)**

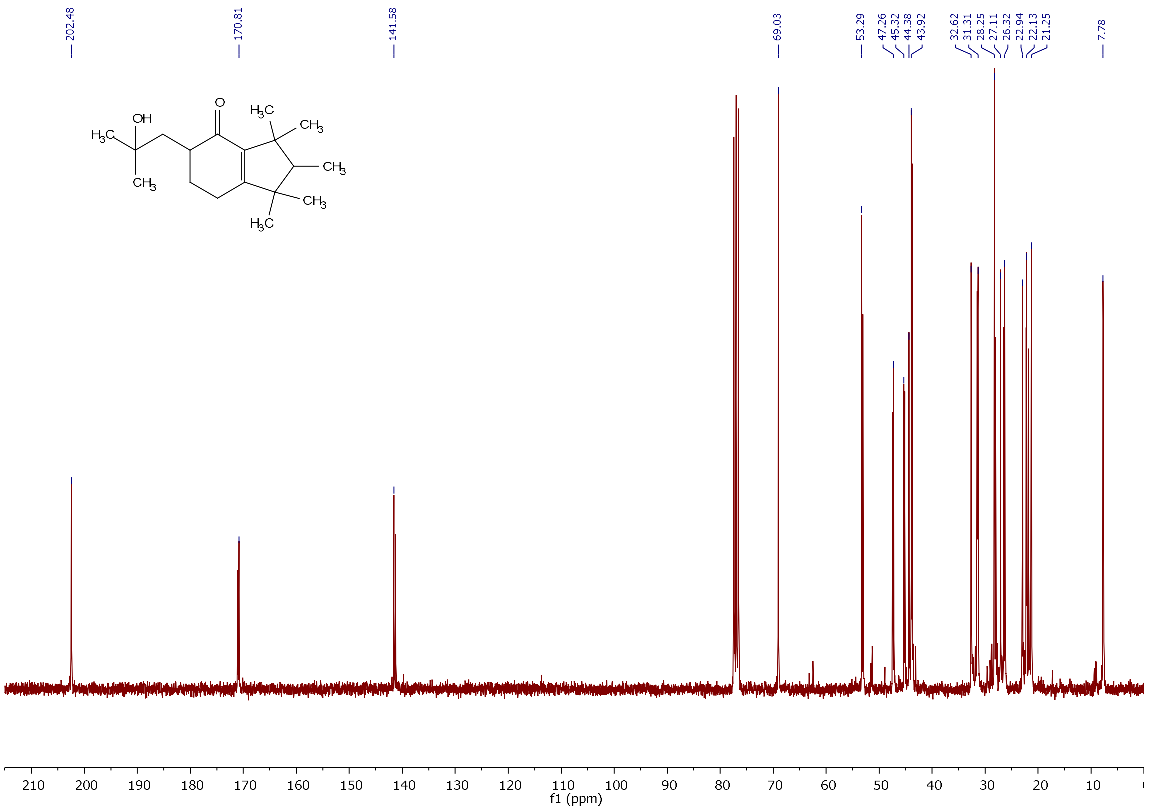

DEPT(75 MHz, Chloroform-*d*) **(4b)**

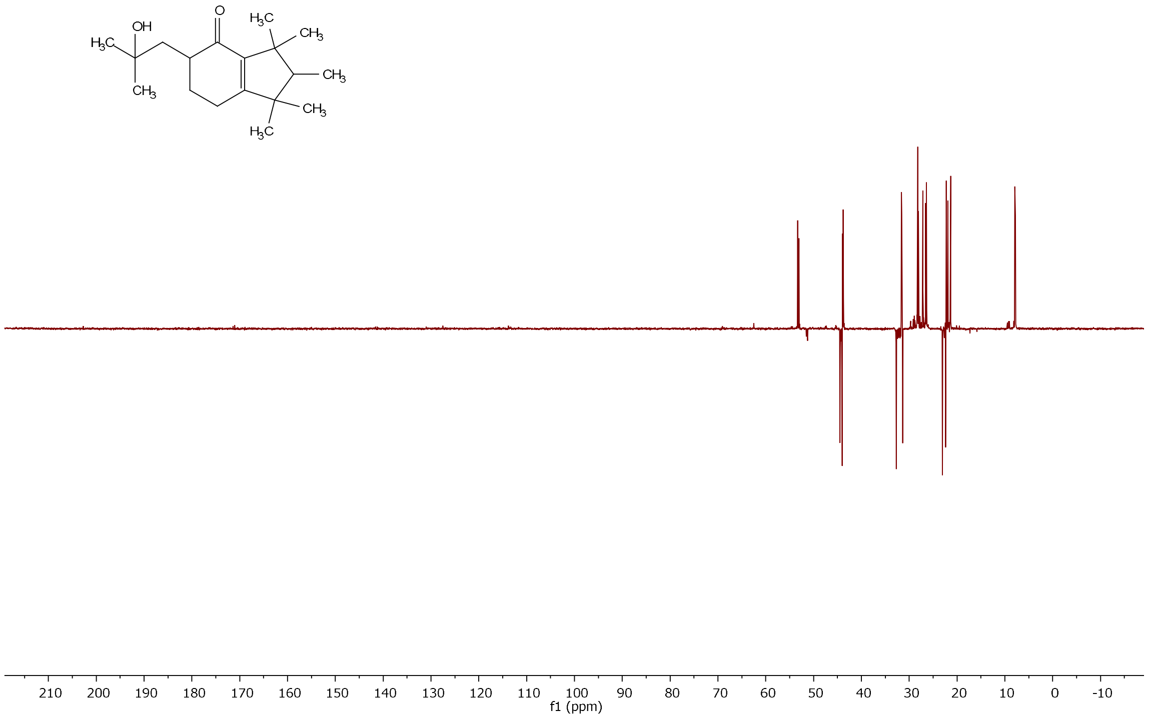

^{1}H (300 MHz, Chloroform-*d*) **NMR (5)**

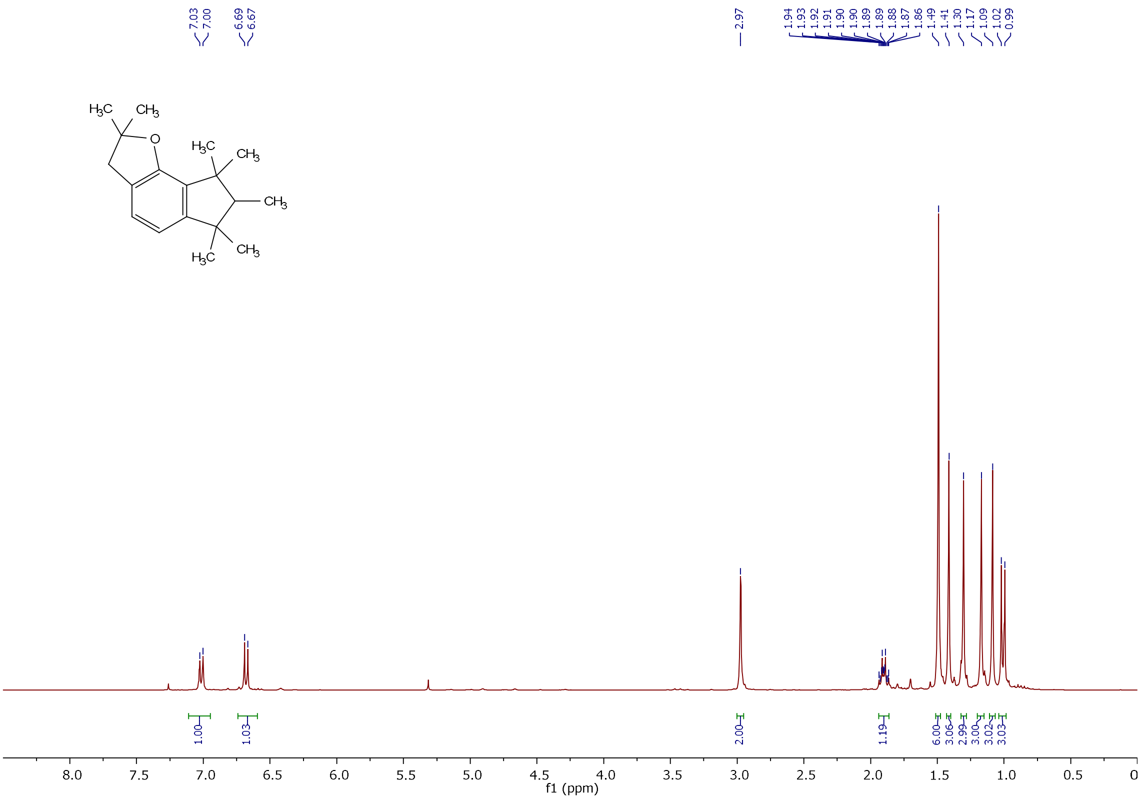

^{13}C{1H}(75 MHz, Chloroform-*d*) **NMR (5)**

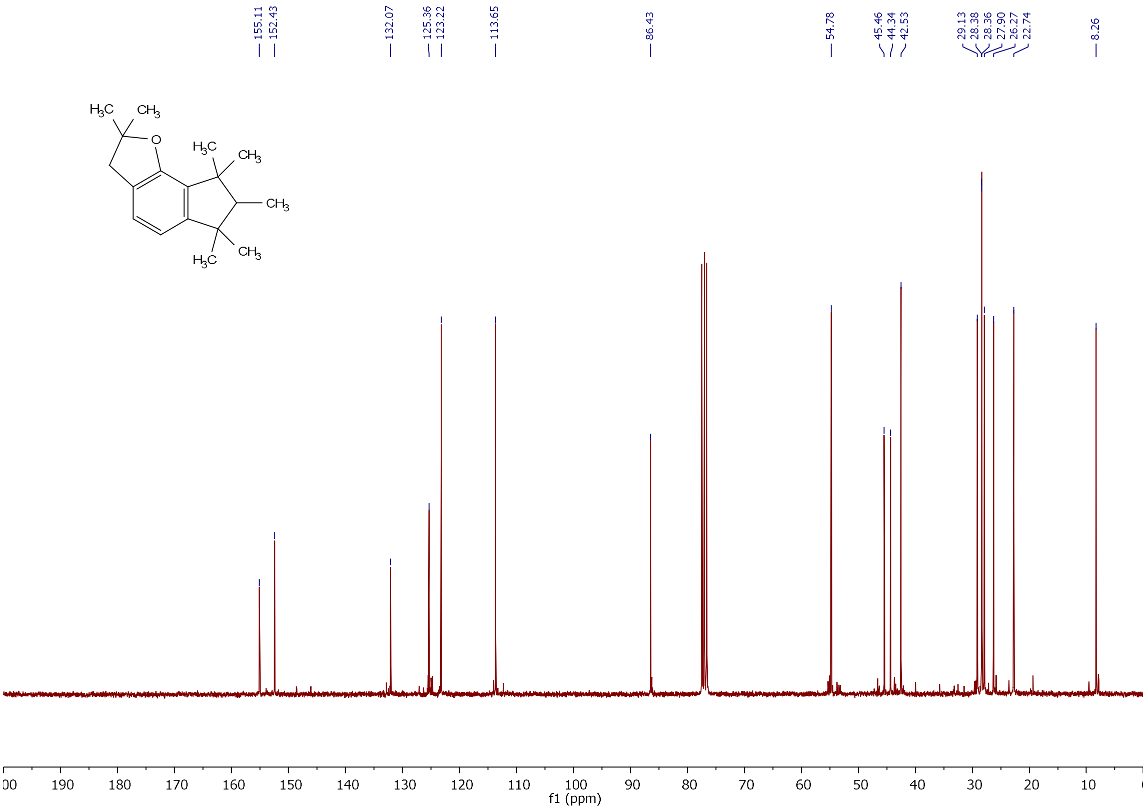

DEPT(75 MHz, Chloroform-*d*) **(5)**

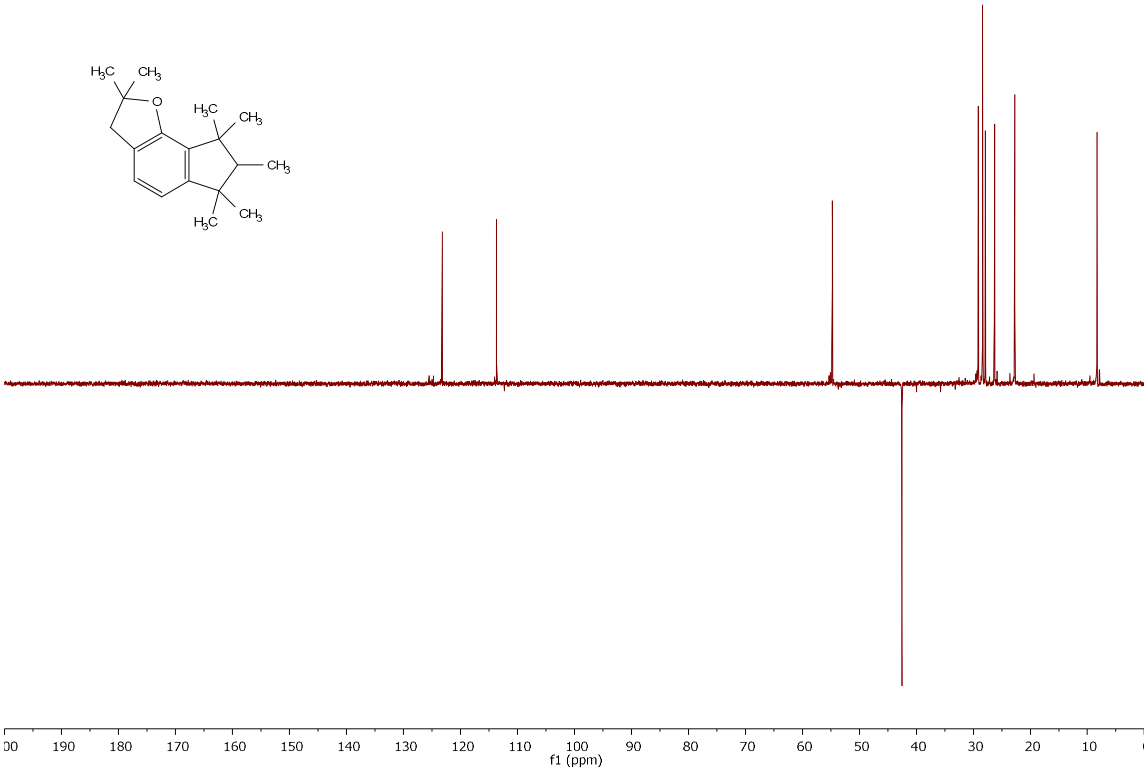

^{1}H (401 MHz, Chloroform-*d*) **NMR (6)**

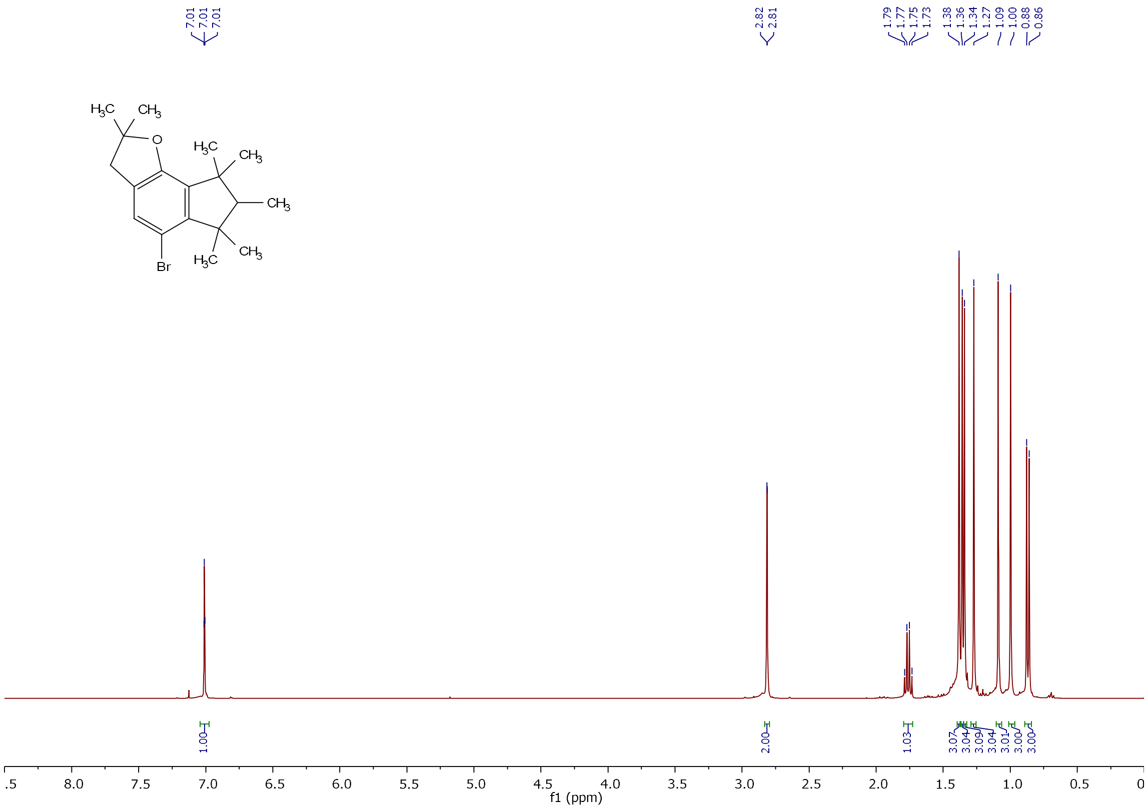

^{13}C{1H} (101 MHz, Chloroform-*d*) **NMR (6)**

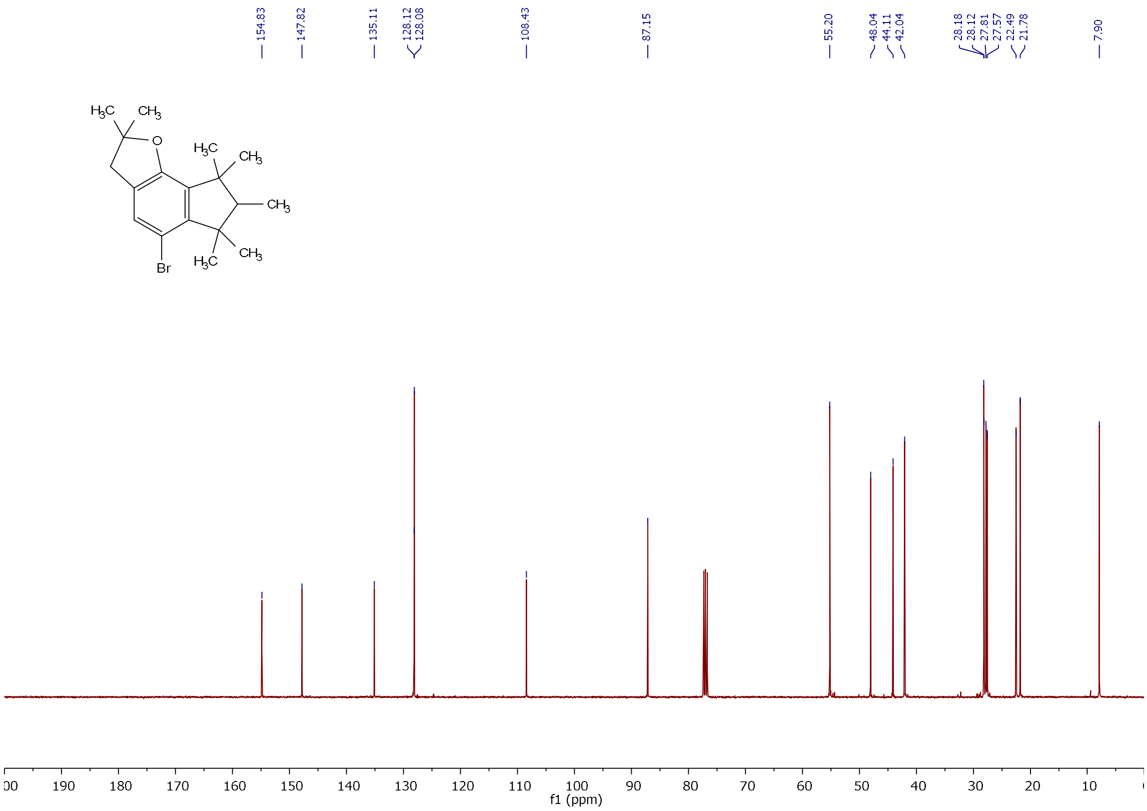

DEPT(101 MHz, Chloroform-*d*) **(6)**

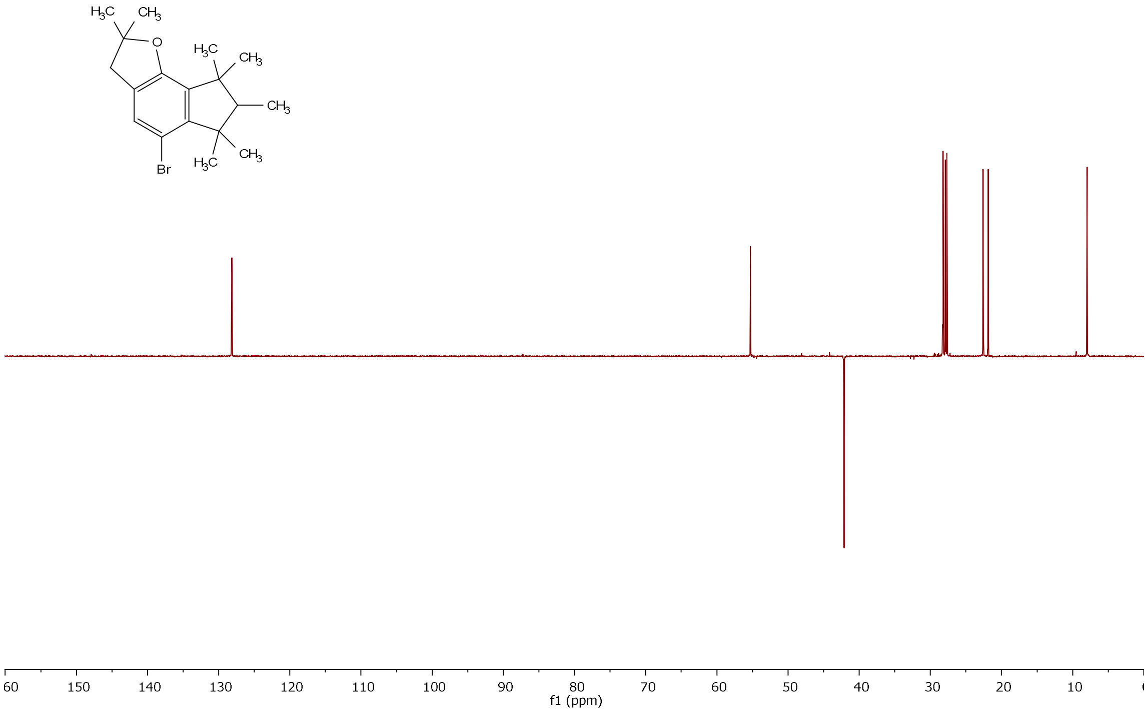

^{1}H (401 MHz, Chloroform-*d*) **NMR (2a)**

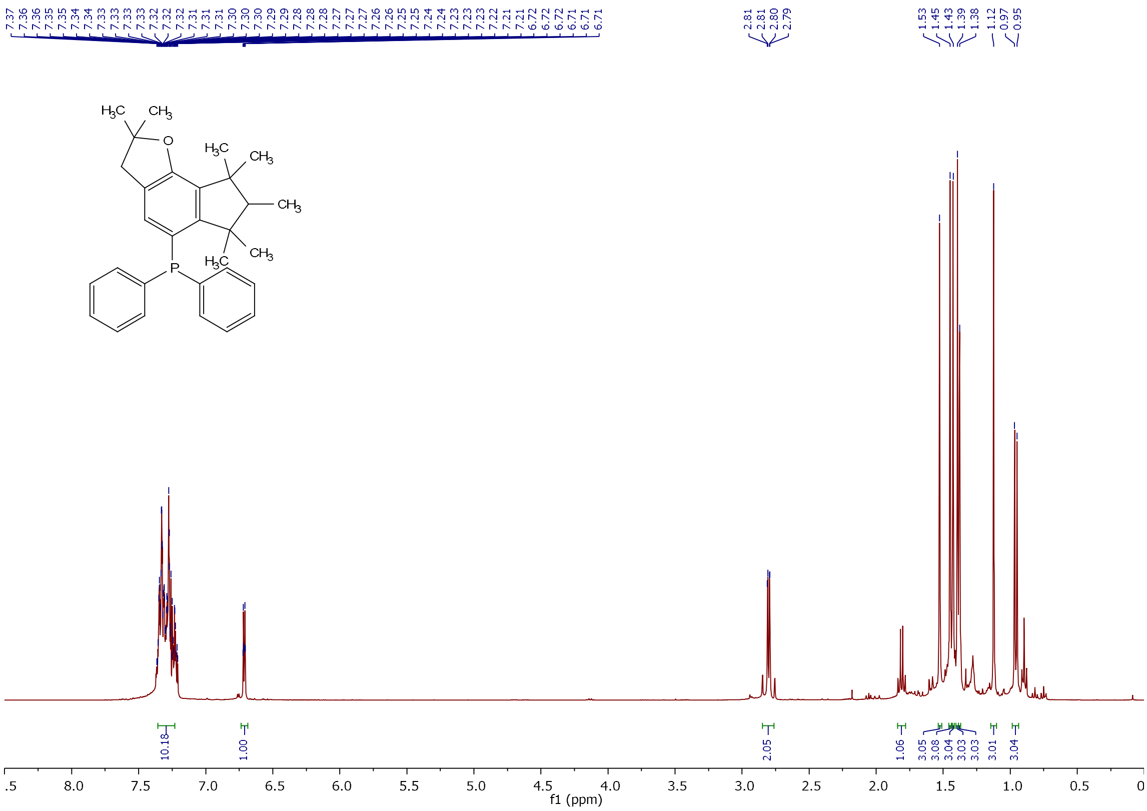

^{31}P (162 MHz, Chloroform-*d*) **NMR (2a)**

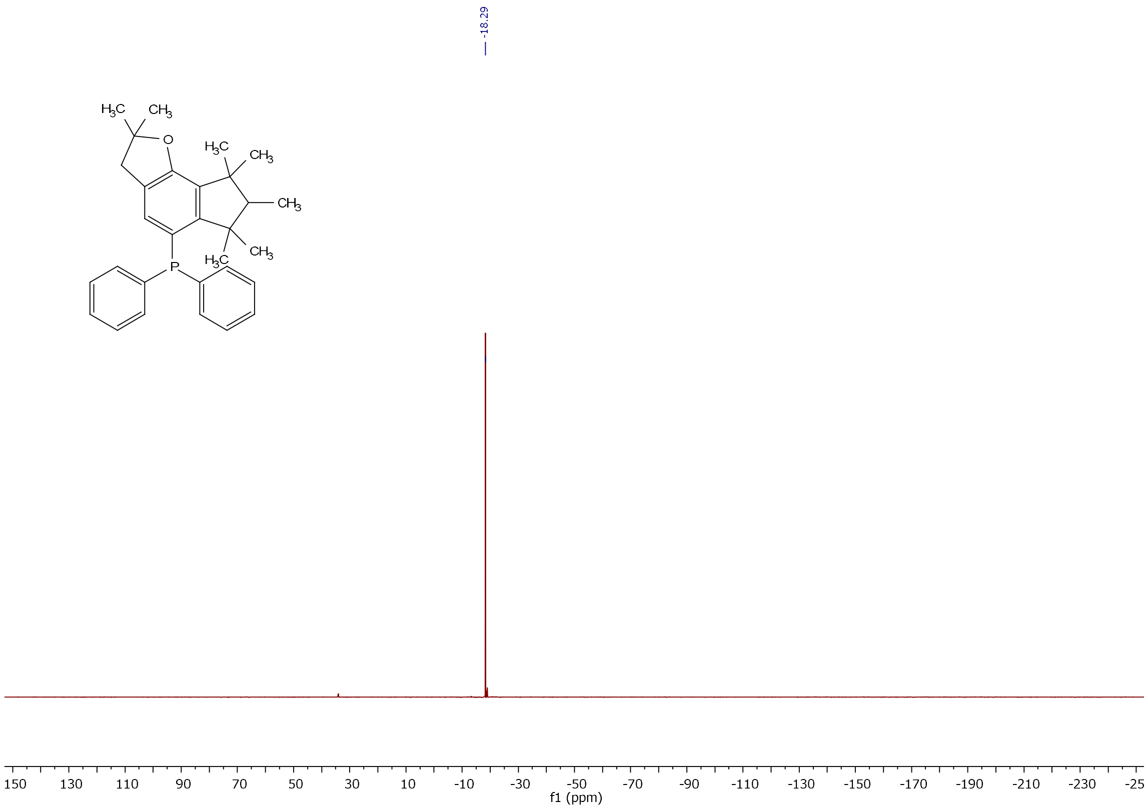

^{13}C{1H} (101 MHz, Chloroform-*d*) **NMR (2a)**

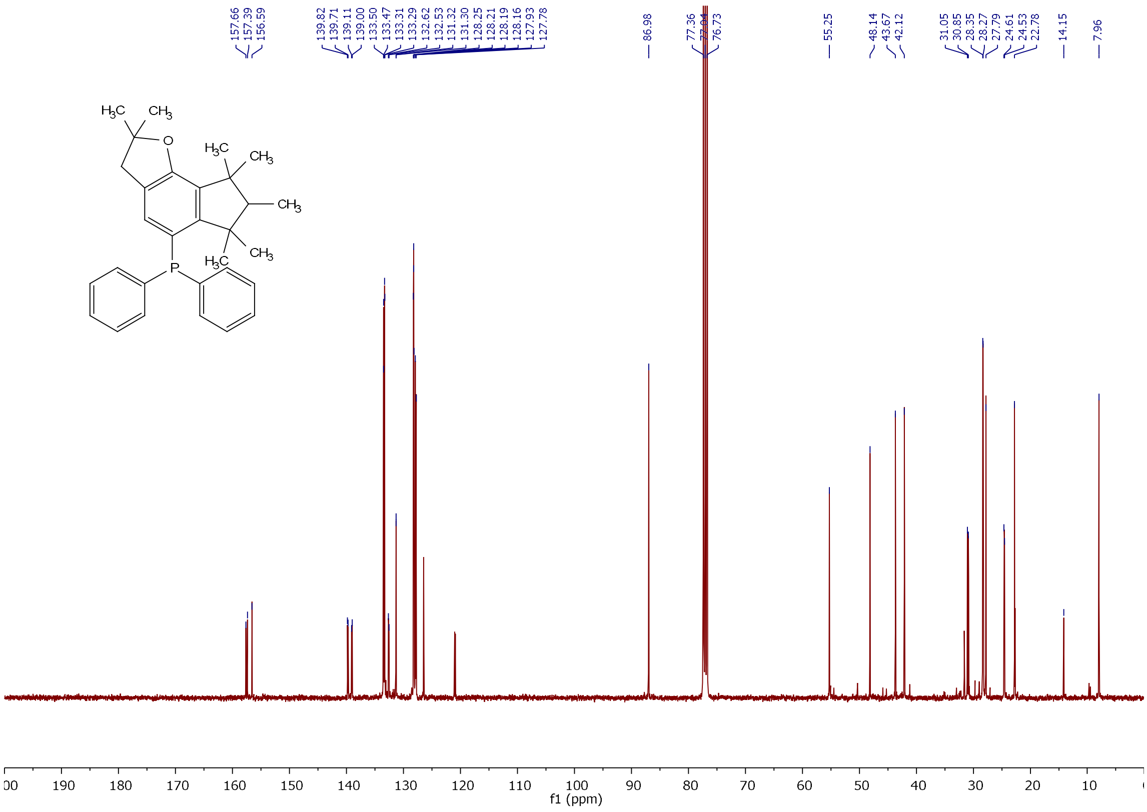

DEPT (101 MHz, Chloroform-*d*) **(2a)**

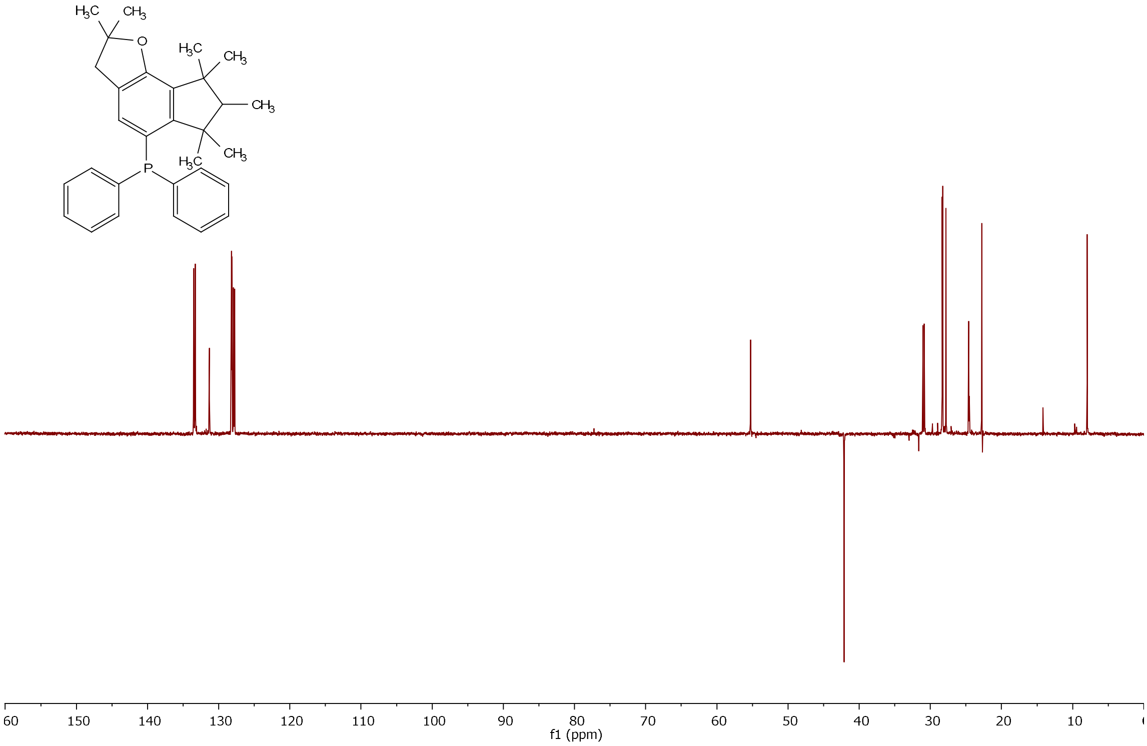

^{1}H (401 MHz, Chloroform-*d*) **NMR (2b)**

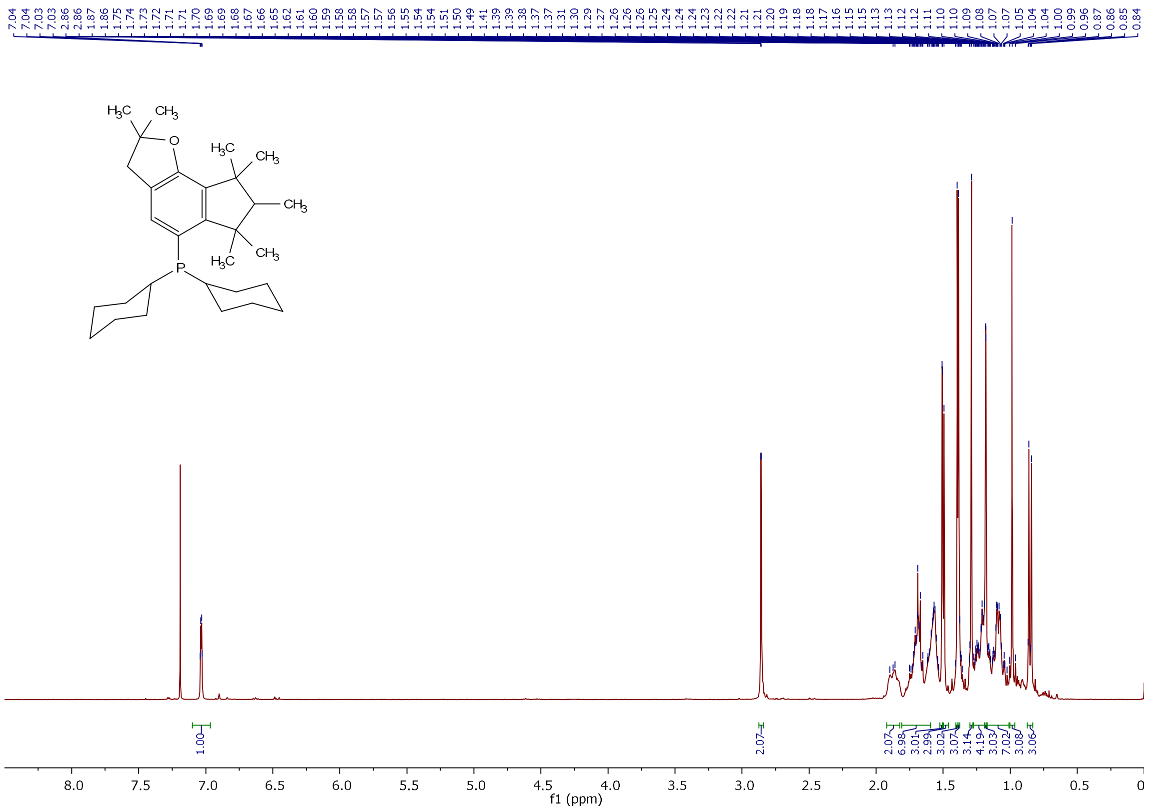

^{31}P (162 MHz, Chloroform-*d*) **NMR (2b)**

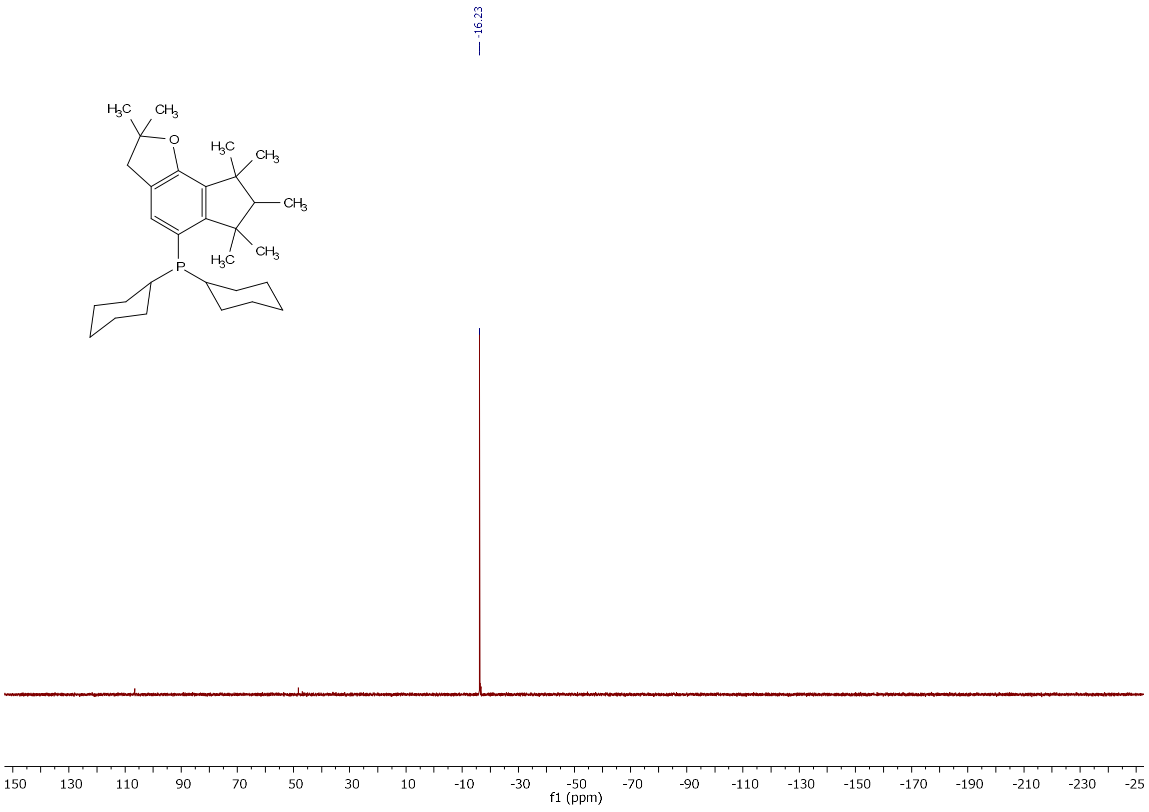

^{13}C{1H} (101 MHz, Chloroform-*d*) **NMR (2b)**

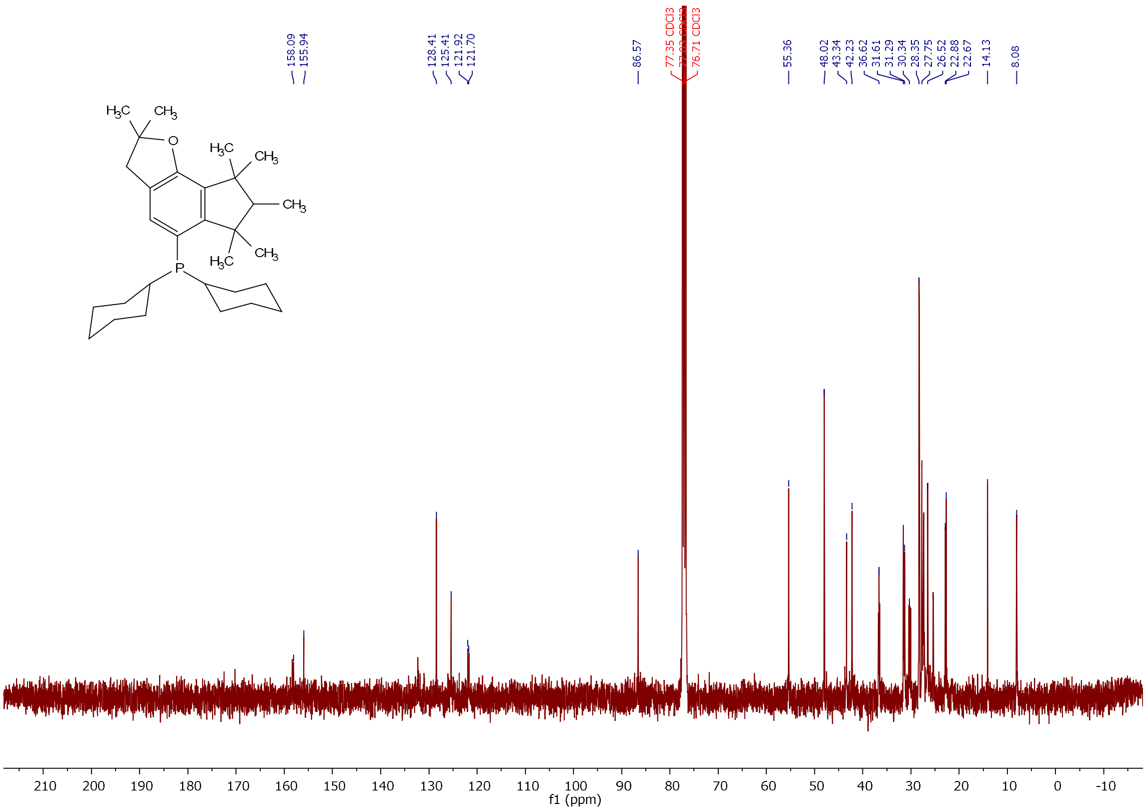

DEPT (101 MHz, Chloroform-*d*) **(2b)**

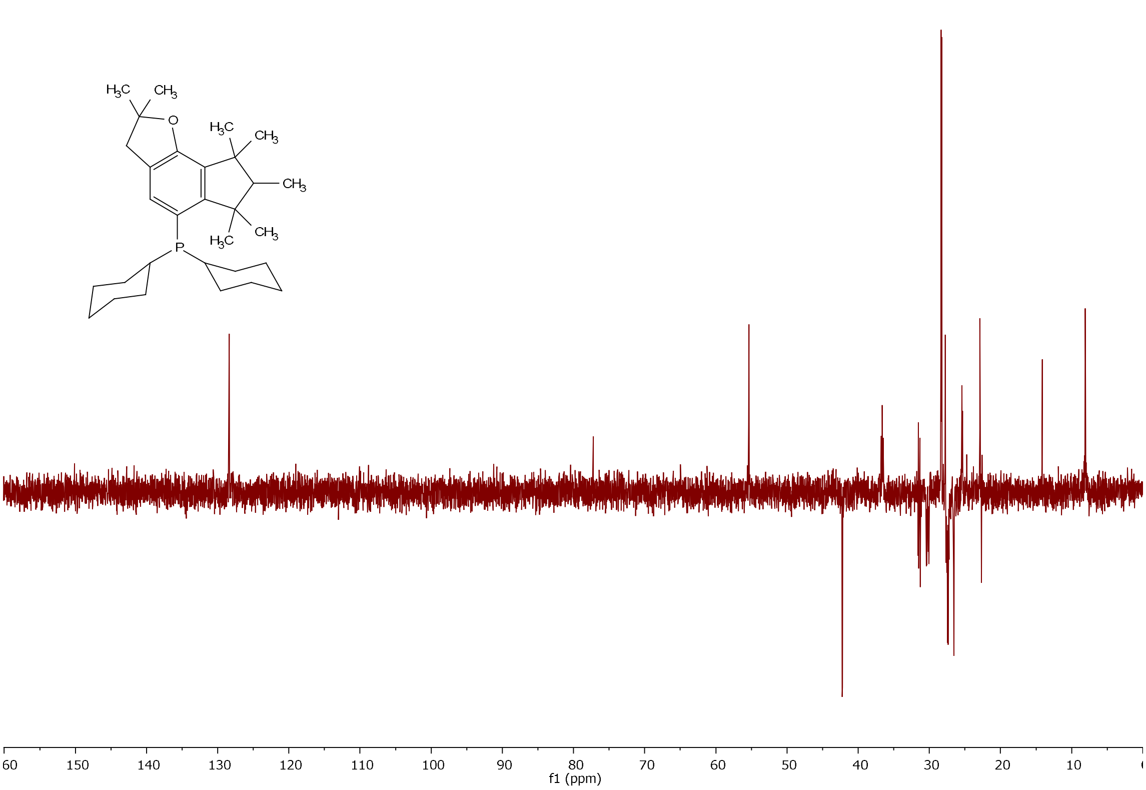

^{1}H (401 MHz, Chloroform-*d*) **NMR ($Pd(OAc)_2(2a)_2$)**

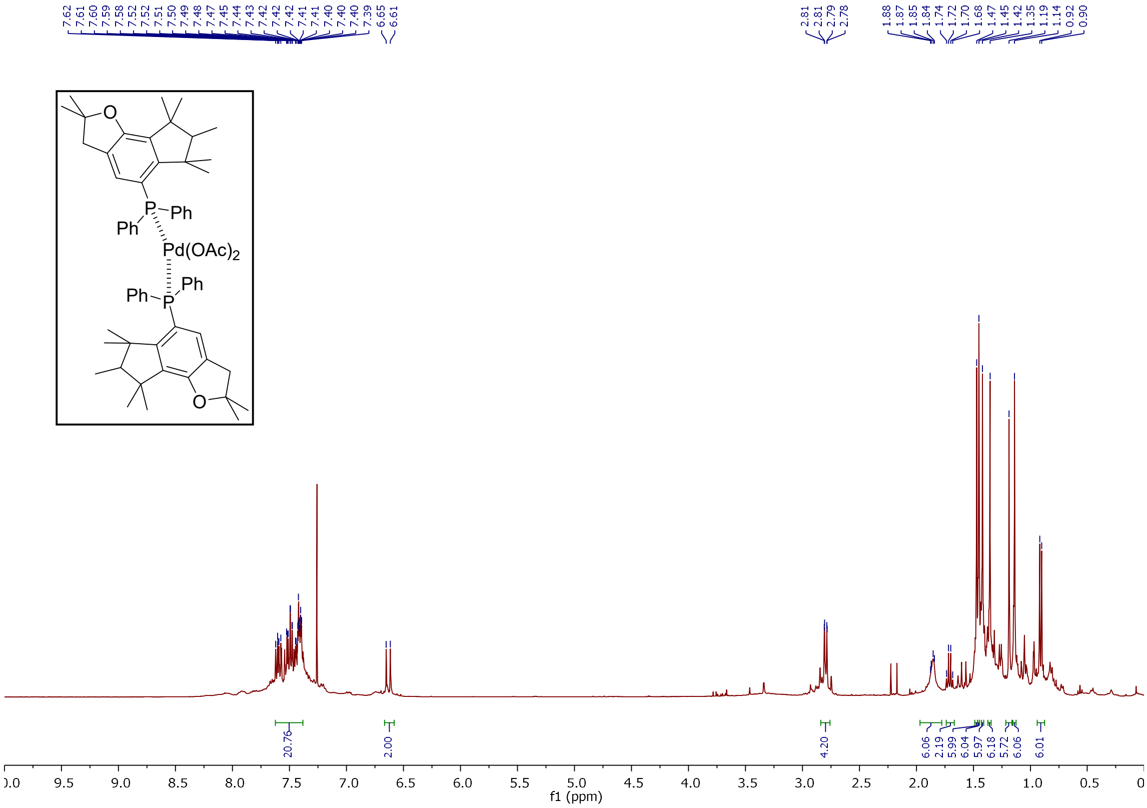

^{31}P (162 MHz, Chloroform-*d*) **NMR ($Pd(OAc)_2(2a)_2$)**

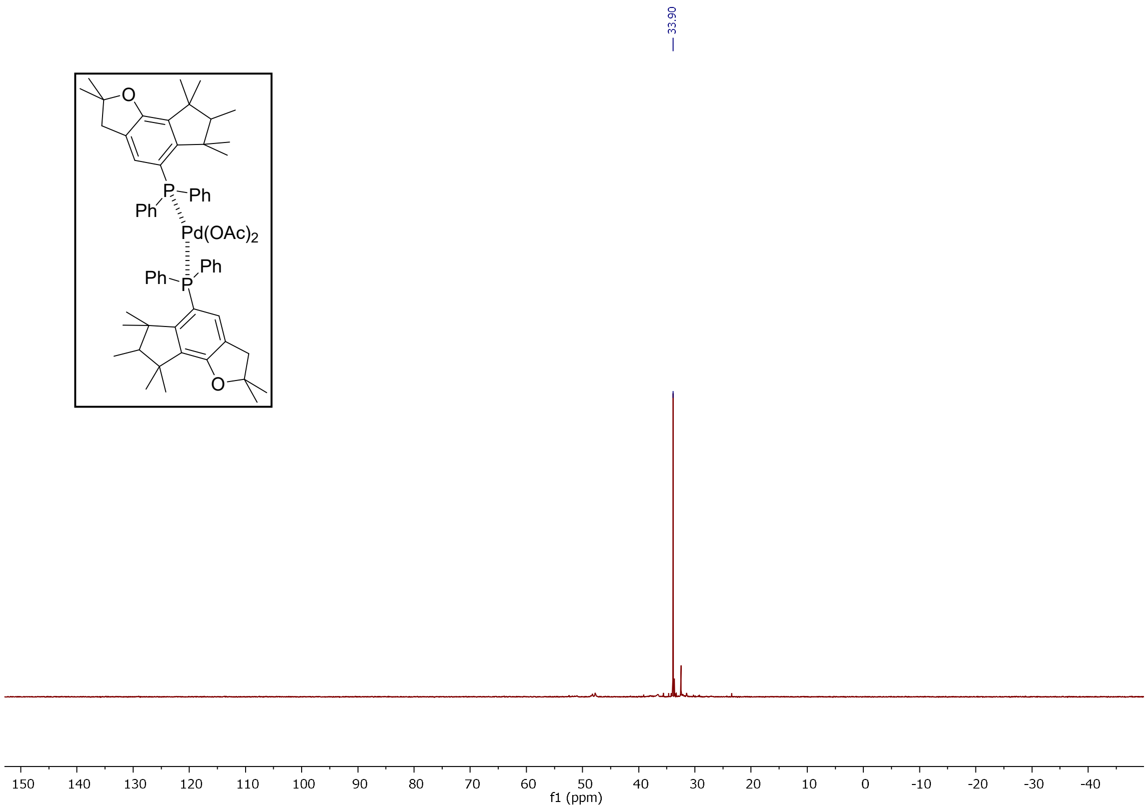

^{13}C{1H} (101 MHz, Chloroform-*d*) **NMR (Pd(OAc)$_2$(2a)$_2$)**

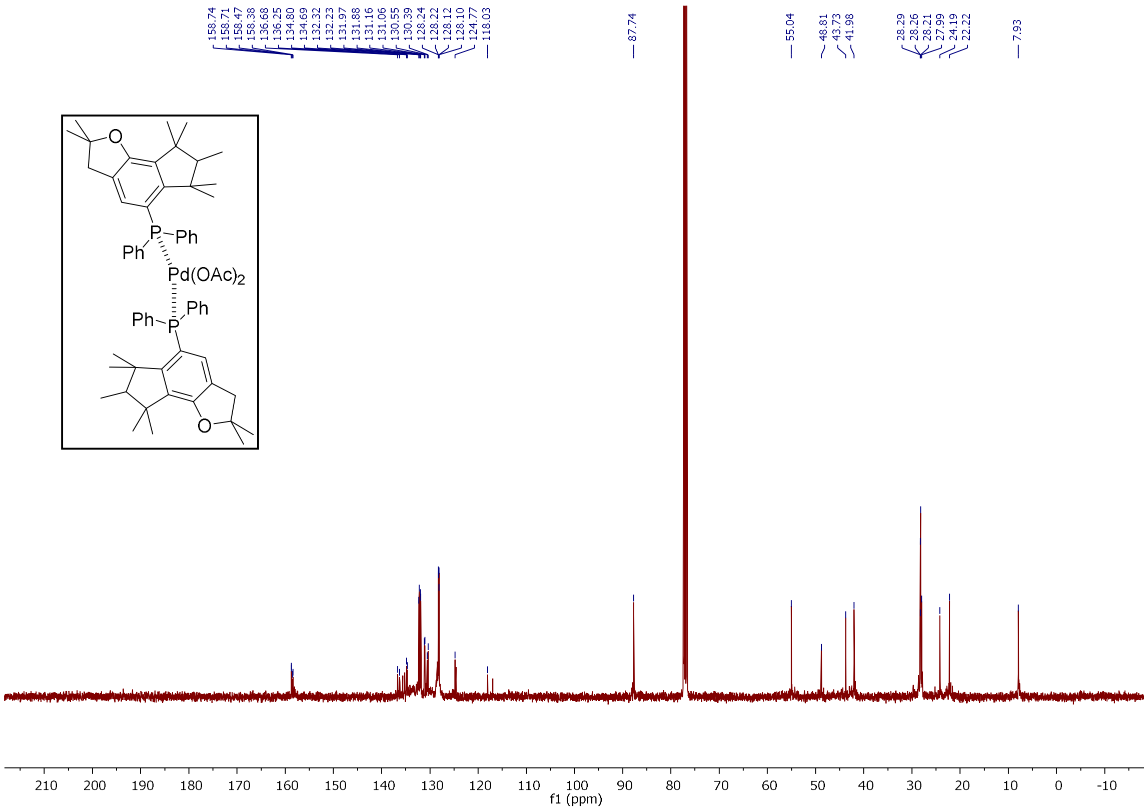

DEPT (101 MHz, Chloroform-*d*) **NMR (2a) (Pd(OAc)$_2$(2a)$_2$)**

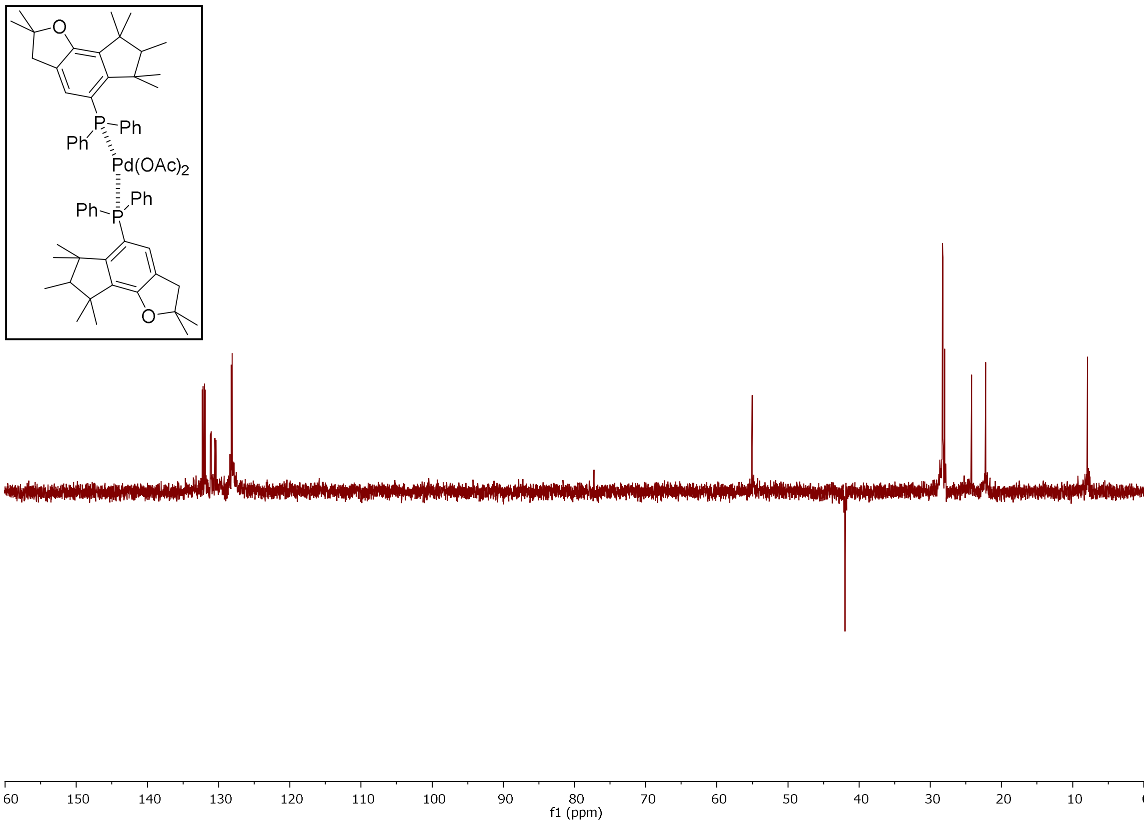

FT-IR copies.

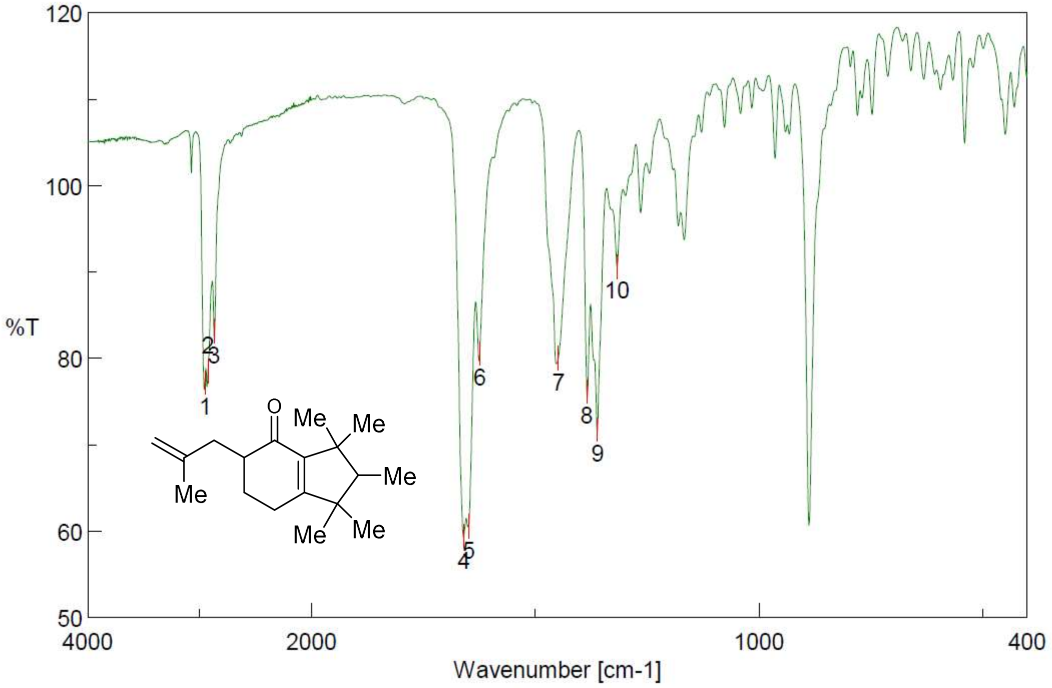

120
100
80
60
50
%T
4000
2000
1000
400
Wavenumber [cm-1]
1
2
3
4
5
6
7
8
9
10
O
Me
Me
Me
Me
Me
Me

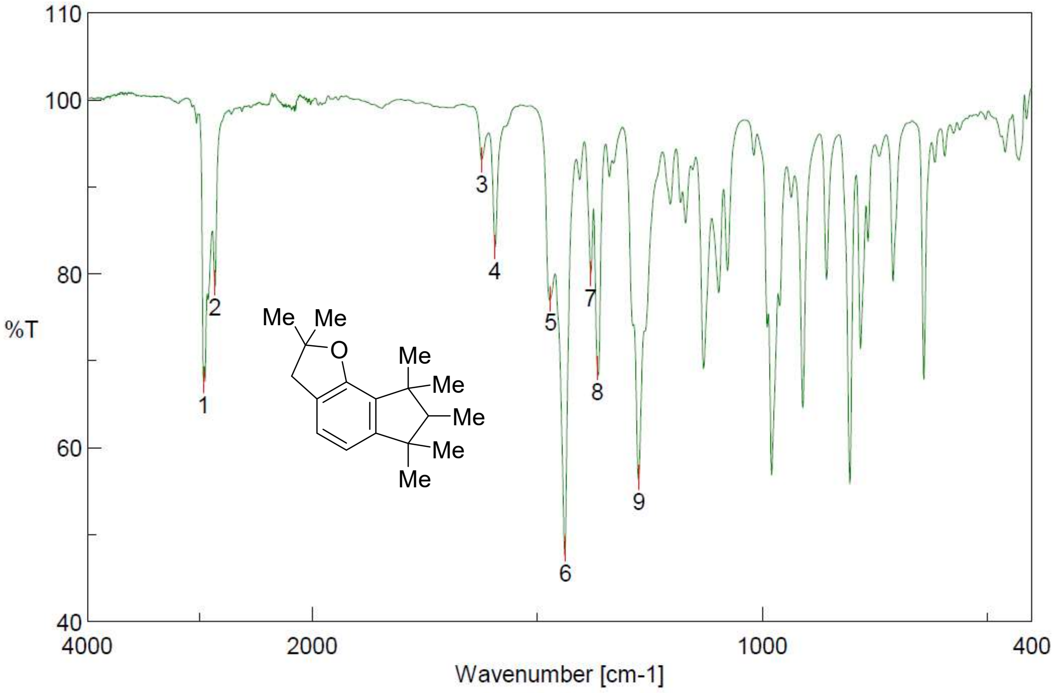

110
100
80
60
40
%T
4000
2000
1000
400
Wavenumber [cm-1]
1
2
3
4
5
6
7
8
9
Me
Me
O
Me
Me
Me
Me
Me

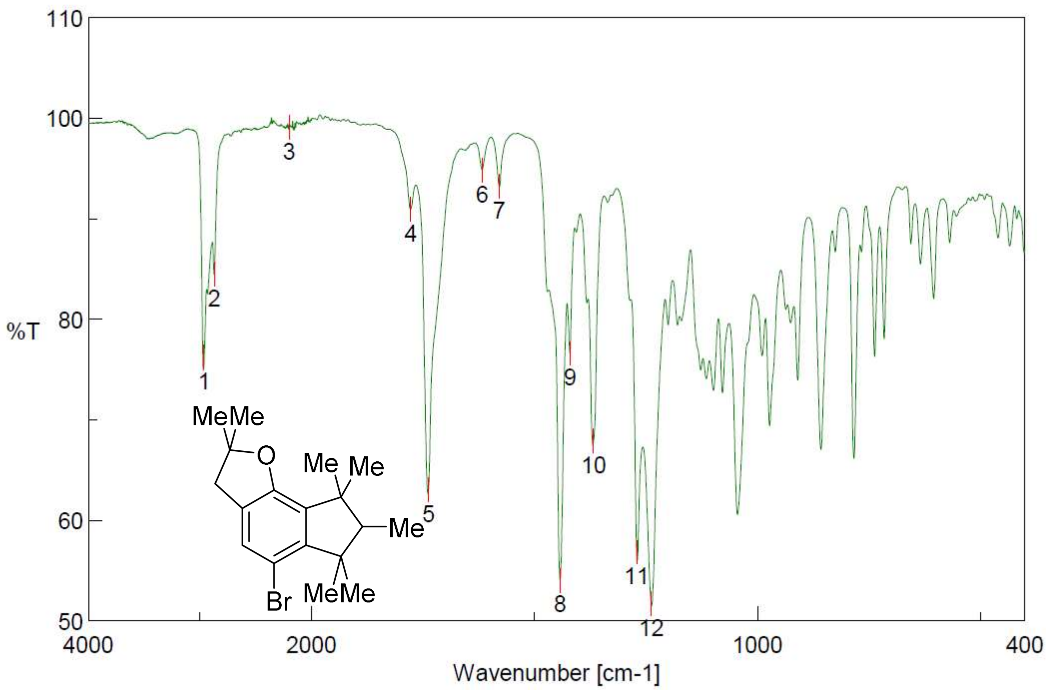
110
100
80
60
50
%T
4000
2000
1000
400
Wavenumber [cm-1]
1
2
3
4
5
6
7
8
9
10
11
12
MeMe
O
Me
Me
Me
Br
MeMe

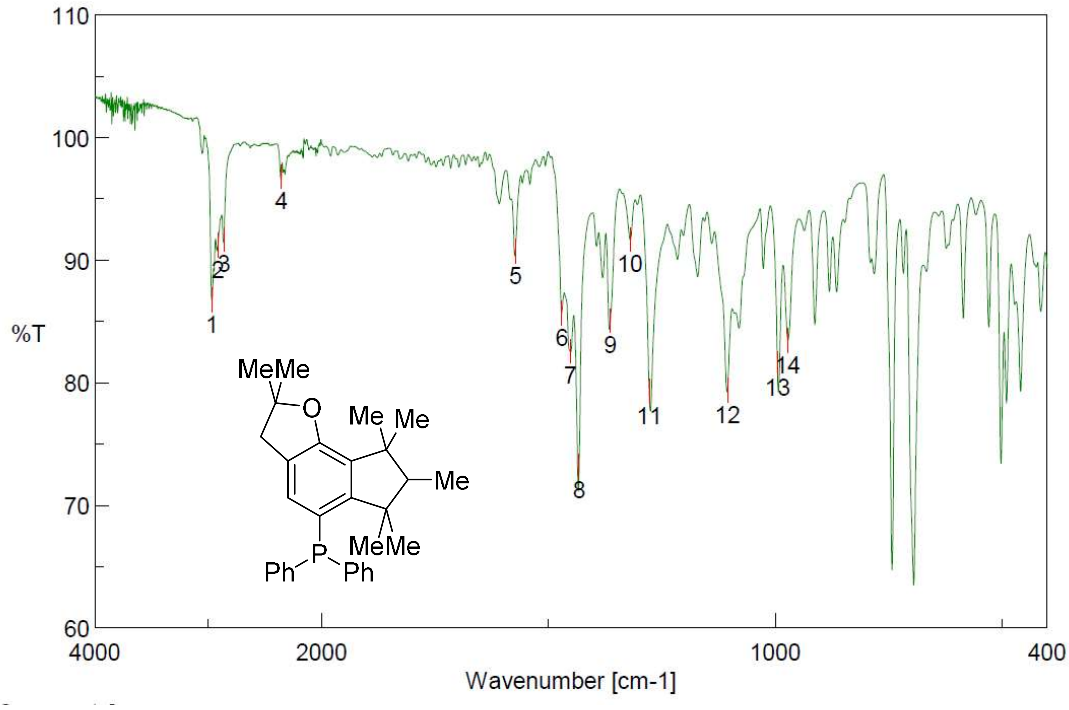
110
100
90
80
70
60
%T
4000
2000
1000
400
Wavenumber [cm-1]
1
2
3
4
5
6
7
8
9
10
11
12
13
14
MeMe
O
Me
Me
Me
MeMe
P
Ph
Ph

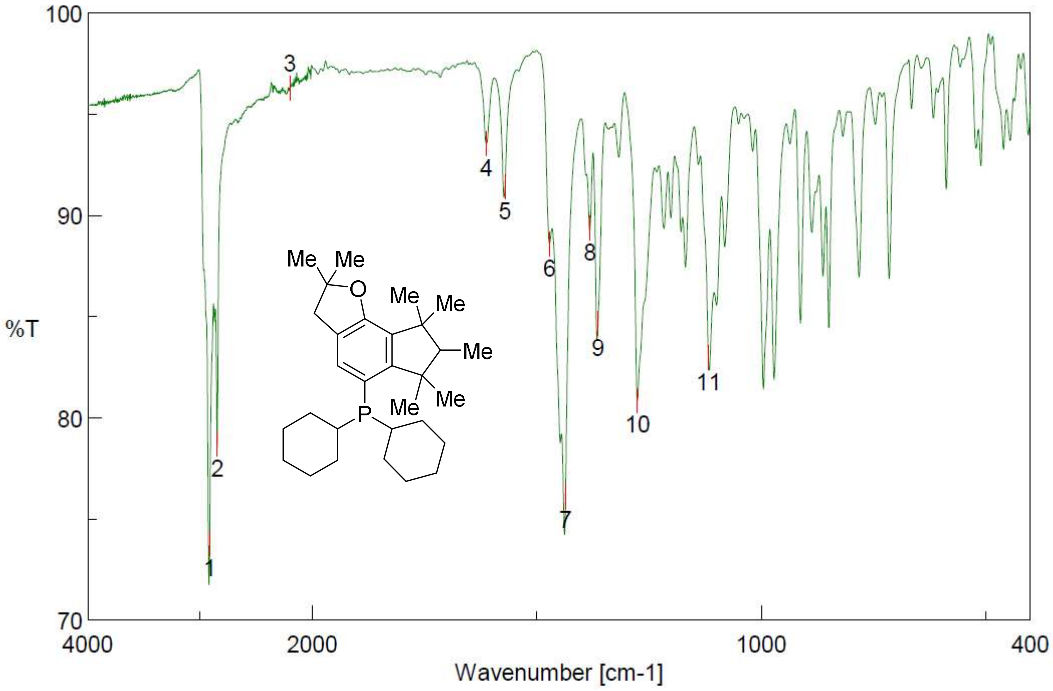
100
90
%T
80
70
4000
2000
1000
400
Wavenumber [cm-1]
1
2
3
4
5
6
7
8
9
10
11
Me
Me
O
Me
Me
Me
Me
Me
P

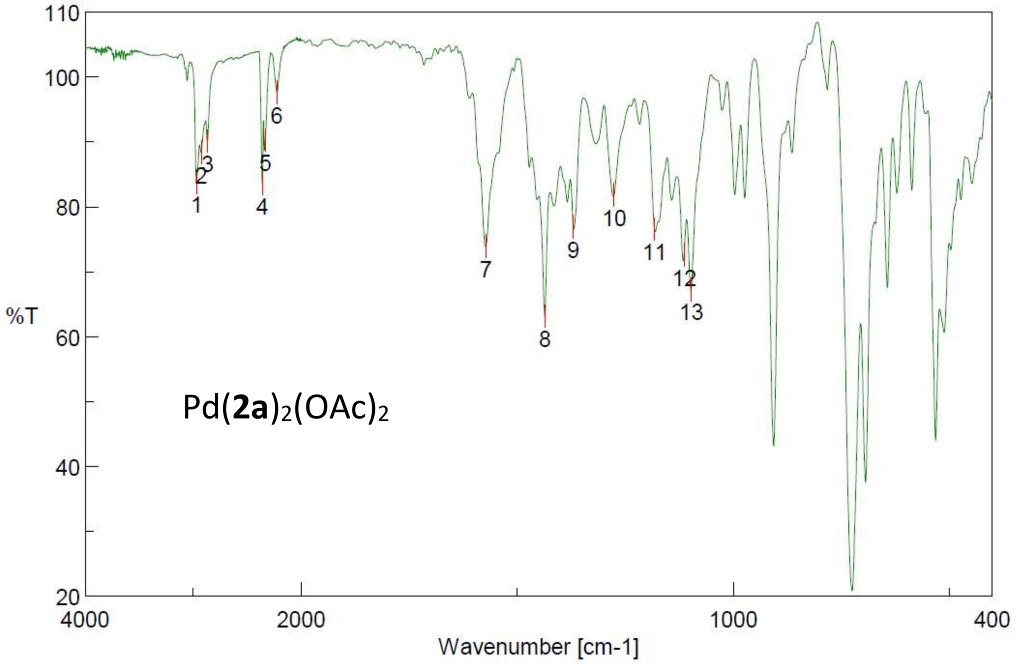
110
100
80
%T
60
40
20
4000
2000
1000
400
Wavenumber [cm-1]
1
2
3
4
5
6
7
8
9
10
11
12
13
Pd(**2a**)2(OAc)2

Summary of crystallographic data.

Compound **6**	Compound **2a**	Compound **2b-oxide**
$C_{18}H_{25}BrO$	$C_{30}H_{35}OP$	$C_{30}H_{47}O_2P$
Mr = 337.29	*Mr* = 442.55	*Mr* = 470.64
Triclinic, *P*1	Monoclinic, *C*2/*c*	Monoclinic, *Ia*
a = 5.9342 (5) Å	*a* = 21.5712 (18) Å	*a* = 11.1537 (8) Å
b = 10.3961 (10) Å	*b* = 11.2764 (8) Å	*b* = 20.5119 (15) Å
c = 13.8224 (13) Å	*c* = 42.689 (3) Å	*c* = 12.4253 (14) Å
α = 102.532 (4)°	β = 103.558 (3)°	β = 106.913 (3)°
β = 93.986 (4)°	-	-
γ = 96.962 (4)°	-	-
V = 822.24 (13) Å^3	*V* = 10094.5 (14) Å^3	*V* = 2719.8 (4) Å^3
Z = 2	*Z* = 16	*Z* = 4
F(000) = 352	*F*(000) = 3808	*F*(000) = 1032
*D*x = 1.362 Mg m^{-3}	*D*x = 1.165 Mg m^{-3}	*D*x = 1.149 Mg m^{-3}
Mo *K*α radiation, λ = 0.71073 Å	Mo *K*α radiation, λ = 0.71073 Å	Mo *K*α radiation, λ = 0.71073 Å
Cell parameters from 9765 reflections	Cell parameters from 9941 reflections	Cell parameters from 9971 reflections
θ = 3.5–30.5°	θ = 2.4–26.5°	θ = 2.9–31.3°
μ = 2.49 mm^{-1}	μ = 0.13 mm^{-1}	μ = 0.13 mm^{-1}
T = 100 K	*T* = 100 K	*T* = 100 K
Prism, translucent colourless	Plate, clear colourless	Block, clear colourless
0.19 × 0.11 × 0.09 mm	0.23 × 0.13 × 0.05 mm	0.29 × 0.17 × 0.10 mm

References.

(S1) Chadwick A., T. Steric Effects of Phosphorus Ligands in Organometallic Chemistry and Homogeneous Catalysis. *Chem. Rev.* **1977**, *77* (3), 314-348. DOI: 10.1021/cr60307a002.

(S2) Jover, J.; Cirera, J. Computational Assessment on the Tolman Cone Angles for P-Ligands. *Dalt. Trans.* **2019**, *48* (40), 15036-15048. DOI: 0.1039/c9dt02876e.

(S3) Michalik, D.; Firdoussi, L.-E.; Beller, M. Telomerization and dimerization of isoprene by in situ generated palladium-carbene catalysts. *J. Organomet. Chem.* **2007**, *692*, 4737-4744. DOI: 10.1016/j.jorganchem.2007.06.039.
